# Supplementary material for: Emotional event perception is related to lexical complexity and emotion knowledge
Source: Commun Psychol. 2023 Dec 18;1:45. doi: 10.1038/s44271-023-00039-4 (PMC11332234; doi:10.1038/s44271-023-00039-4)
Supplement: Supplementary file 1 — Supplemental Material [file 44271_2023_39_MOESM1_ESM.pdf]

Supplementary Information

This appendix contains supplementary information on the following topics:

I. Supplementary Methods and Results

a. Supplementary Note.1 Stimuli construction (p. 2)
b. Supplementary Note 2. Debriefing questions following emotion segmentation task (p. 6)
c. Supplementary Note 3. Semantic Agreement Score (SAS) and Semantic Distinctiveness Score (SDS) calculation and analysis (p. 14)
d. Supplementary Note 4: Additional analysis with Psychological Well-Being Scale (PWB) and Autism Spectrum Quotient - 10 items (AQ-10) (p. 24)
e. Supplementary Note 5: Representational Similarity Analysis (RSA) task and analysis (p. 27)
f. Supplementary Note 6: Exploratory analysis using Signal Detection Theory (p.31)
g. Supplementary Note 7: Exploratory analysis on emotion labels and segmentation frequency (p.54)

III. Supplementary References (p. 59)

Supplementary Note 1. Stimuli construction

To our knowledge, there are no existing sets of documentary clips specifically designed for use as emotion perception task stimuli. Hence, we constructed a set of documentary clip stimuli with the following procedure.

We first used the advanced search feature of IMDb and searched for color documentaries released between 2016/1/1 and 2021/1/1 (within the last 5 years at the time the set was constructed), with user ratings between 9 to 10. We examined each documentary by examining its synopsis and trailer and selected documentaries that are observational documentaries that center on human subjects and attempt to capture everyday life unobtrusively. This search yielded five potential documentaries: *American Factory (2019)*, *The Ugly Model (2019)*, *This Might Hurt (2019)*, *Take Me Home (2020)*, and *The Invisible Class (2020)*. Among these, *The Invisible Class (2020)* and *The Ugly Model (2019)* were excluded due to inaccessibility of the films.

Given the limited set of films yielded by this process, we opted to expand the list of documentaries. We conducted a second round of search for color documentaries released between 2011/1/1 and 2021/1/1, with user ratings between 8 and 10, and the rating count of at least 1000. The additional parameter of rating count was added to further ensure the quality of the documentary. This search yielded four additional potential documentaries: *Free Solo (2018)*, *The Red Pill (2016)*, *Minding the Gap (2018)*, and *Rewind (2019)*. Among these, *Rewind (2019)* was excluded for its highly evocative content.

Finally, for a third round of search, we adopted the same parameters as the second search, only with the rating range expanded to between 7 and 10. This search yielded six additional potential documentaries: *Unrest (2017)*, *A Secret Love (2020)*, *Rich Hill (2014)*, *The Mask You Live In (2015)*, *Twinster (2015)*, and *Extremis (2016)*.

In addition, we also conducted ad hoc search and screening of the resulting films. Among the ad hoc films screened, *Found (2021)*, *For Ahkeem (2017)*, *Hot Girl Wanted (2015)*, *The Surgeon's Cut (2021)*, and *Foster (2018)* were identified as potential sources of stimuli. After three waves of search and these ad hoc additions, we yielded a total of 11 documentaries to choose our clips from.

In line with our selection criteria for film stimuli used in Study 1, we use the following standards to select clips from the 12 documentaries: 1) The clips should be 3 minutes or shorter, with ideally a single person on camera (two people were acceptable when the clip contained minimal shots of other individuals). 2) The person in the clips should ideally be engaged in live action, though interviews may also be acceptable on a case-by-case basis. 3) The clip should ideally demonstrate a variety of dynamic emotions. In other words, one should be able to identify multiple emotions or varied intensity and dynamics of emotions within the clip.

In-lab screening sessions were held where the researchers and RAs viewed and discussed these criteria for selecting clips using *Extremis (2016)* as an example. After the group screening, the researchers and RAs each watched one or two films and selected 3-4 clips from each, following the guideline illustrated above. Two additional screenings were held where the selected clips were viewed collectively and discussed. Among all the clips, 9 were deemed to be of high quality with respect to the guideline and controlling for reappearing characters. These include 2 clips from *Extremis (2016)* (77s, mainly featuring one middle-aged White female; 67s, mainly featuring one middle-aged Black male and one young South East Asian female), 1 clip from *Twinster (2015)* (166s, mainly featuring two East Asian teenaged girls), 1 clip from *A Secret Love (2020)* (66s, mainly featuring two White males and two White females, all older adults), 1 clip from *Unrest (2017)* (91s, featuring one young Black male and one young Black

female), 2 clips from *Minding the Gap (2018)* (74s, featuring one young White male and one young White female; 111s, featuring one Black teenaged boy), 1 clip from *Found (2021)* (94s, mainly featuring two East Asian teenaged girls), and 1 clip from For *Akheem (2017)* (123s, mainly featuring one teenaged Black male and one teenaged Black female). A pilot study with *N* = 30 via prolific (Due to technical issues, 3 subjects did not have their demographic information recorded. For the remaining 27 subjects, $M_{\text{age}} = 29.29$, $SD_{\text{age}} = 12.7$; 10 male, 16 female, 1 non-binary; 19 identified as White, 1 identified as Black or African American, 3 identified as Asian, 2 identified as Hispanic or Latinx, 1 identified as mixed race, and 1 identified as ‘other’.) was conducted to reaffirm if the clips were identified to contain sufficiently diverse emotions. Analysis of the pilot data showed that across the 9 clips, the median numbers of segmentation for the clips range from 2 to 8.5, and the number of critical events range from 1 to 4. These result suggest that the clips contain sufficiently distinct emotion events for people to identify.

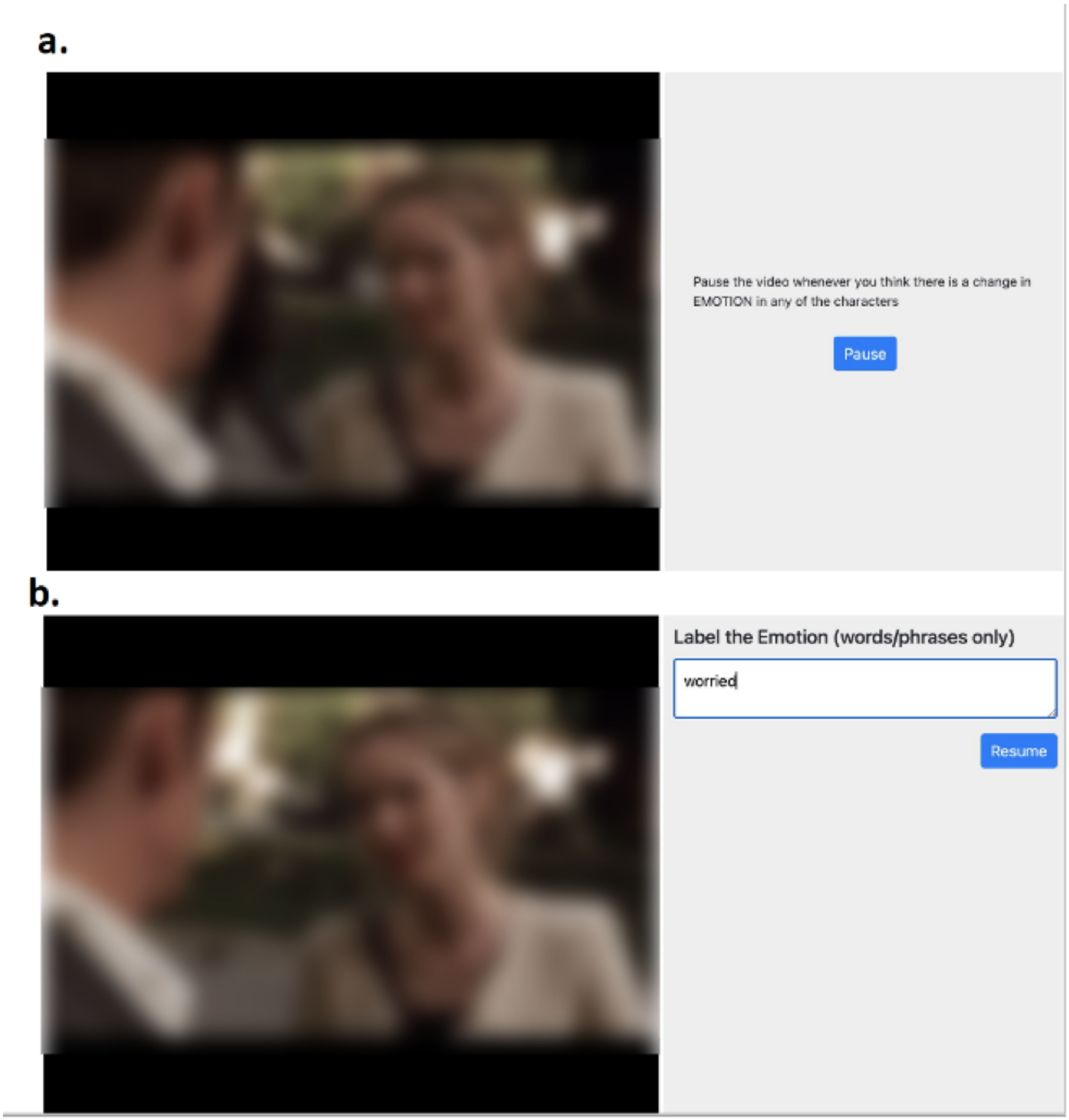

Supplementary Figure 1: **Emotion segmentation task interface. a**, Interface displaying instructions and pause button while the video plays. **b**, Interface displaying space for text entry and resume button while the video pauses.

Supplementary Note 2. Debriefing questions following emotion segmentation task

Debriefing involved five questions after participants completed the task session. Each question and details of the responses are provided below. Raw responses are available in the OSF repository: https://osf.io/zsu7t/?view_only=90f62ea1056c4b4e995514d99fe59e3a

First, we asked participants: "Have you seen any of these films previously? Please click the films that you have watched before?" (options for Study 1: Spotlight (2015); Selena (1997); Where the Heart is (2000); Me Before You (2016); Love Rosie (2014); options for Study 2: Minding the Gap (2018); Unrest (2017); Found (2021); Twinster (2015); Extremis (2016); Found (2021)). For Study 1, 128 (51% of participants) reported that they had seen at least one film. Given this level of familiarity, we examined whether this exposure was related to our primary segmentation performance metric Consensus Event Agreement (CEA). We found that familiarity did not significantly predict segmentation performance (R^2 = .009, $F(247)$ = 3.26, P = .07). For Study 2, 26 (10% of participants) reported that they had seen at least one documentary, and familiarity did not significantly predict segmentation performance represented by CEA (R^2 = .007, $F(252)$ = 2.77, P = .10).

We also asked participants about their understanding of the instructions using the following open-response question: "Did you have any problems with the segmentation tasks? Were you confused about any of the instructions?" Overall, few participants reported confusion about the instructions. When the few participants did report ambiguity in the instructions, they indicated that it was a challenge to distinguish between emotions in the emotion segmentation task and behaviors in the control segmentation task. For instance, some indicated that they did not fully understand the meaning of "behaviors" in the instructions and treated emotion as

behaviors in the control trials. Across two studies, only 10 participants (out of 483, or 2%) reported such challenges, suggesting that the task and instructions were broadly understood.

Given that we instructed participants to segment the films in a fine grained manner (“any change in the characters’ emotions”), we asked participants how finely they segmented as an instruction check: “How finely or coarsely did you feel you were segmenting the film clips by emotion?”(options: 1= Very finely: I paused whenever I saw a small change of emotional unit on screen; Somewhat finely; Neutral; Somewhat coarsely; 5= Very coarsely: I only paused when I saw a significant change of emotion.). In Study 1, participants overall reported a mean of 2.25 (*SD* = 1.09) corresponding to between “Somewhat finely” and “Neutral”. In Study 2, participants overall reported a mean of 2.30 (*SD* = 1.07), falling into the same level of grain. This suggests that participants were following the instructions to segment in a relatively fine-grained manner. We also examined whether this self-reported “grain” was related to the metric of Mean Length of Units (MLU), which reflects how coarsely or finely participants segmented in the task. We did find a relationship between these two measures, as the self-reported “grain” was positively correlated with MLU (Spearman’s ρ = .210; 95% CI, (-0.08, 0.34); P = .0004). The self-reported “grain” also significantly predicted MLU (R^2 = .03, $F(247)$ = 9.13, P = .003). These results suggest that those who reported to have segmented finely indeed segmented in a more finely manner which resulted in shorter segmented units on average. This pattern was replicated in Study 2, where self-reported “grain” was positively correlated with MLU (ρ = .230; 95% CI, (0.11, 0.54); P = .0001) and also predicted MLU (R^2 = .06, $F(252)$ = 16.64, $P < .001$).

We were also interested in whether participants often segmented even when they did not have an immediately accessible semantic label in mind for what they perceived: “Did you pause in the emotion task even when you could not immediately provide a label?” (options: 1=Never;

Rarely; Sometimes; Often; 5=Always). We instructed participants to segment even on occasions when a label was not immediately accessible. In Study 1, participants responded with a mean of 2.95 (*SD* = 0.94) corresponding to between "Rarely" and "Sometimes" on the scale. Similar pattern is observed in Study 2 (*M* = 2.85; *SD* = 1.00). This indicates that in some instances, participants noticed an emotion shift but from their subjective perspective, with labeling followed. It is important to caution that the subjective sense of access to a label does not necessarily speak to the underlying order of cognitive operations.

Finally, we sought to understand what strategies participants may have used to segment the videos: "What strategies/cues did you use to segment the videos by emotion?" (free-response question). Most participants reported that they relied on nonverbal cues (e.g., facial expression, vocal tone, body language) as well as verbal information. Several participants also reported that they relied on an empathic "feeling with" strategy to perceive emotional changes in the characters. Participants did not report relying on structural aspects of the films (e.g., cuts, music) although it is possible that these cues had an influence on perceptions without participants' awareness.

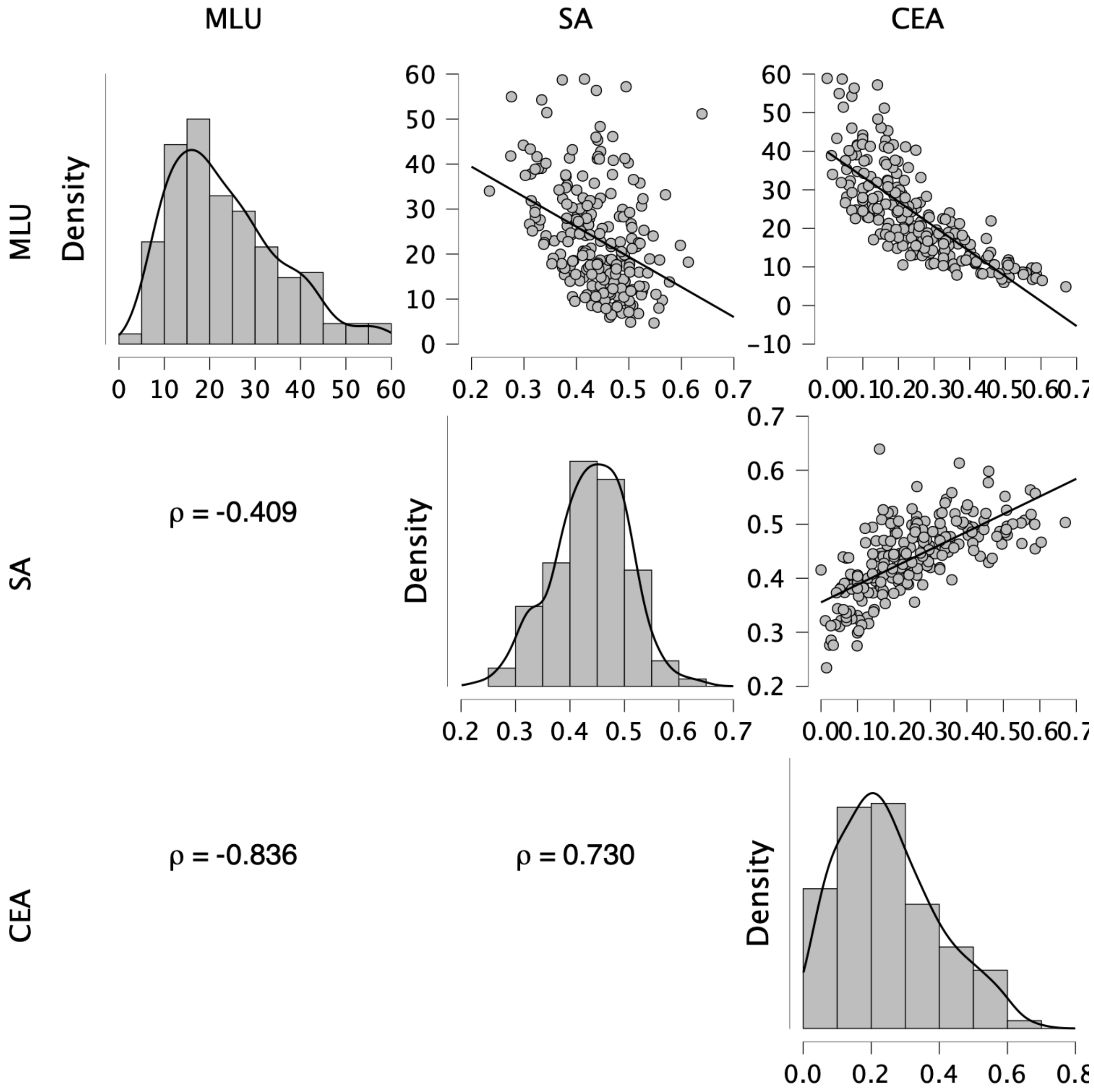

Supplementary Figure 2. **Histogram and scatter plot depicting relationship between emotion segmentation metrics of Study 1**. All three metrics are significantly correlated with each other in the expected direction, with Mean Length of Unit (MLU) negatively correlated with both Segmentation Agreement (SA) and Consensus Event Agreement (CEA) (as better segmentation performance is associated with shorter length of segmentation unit), and SA positively correlated with CEA.

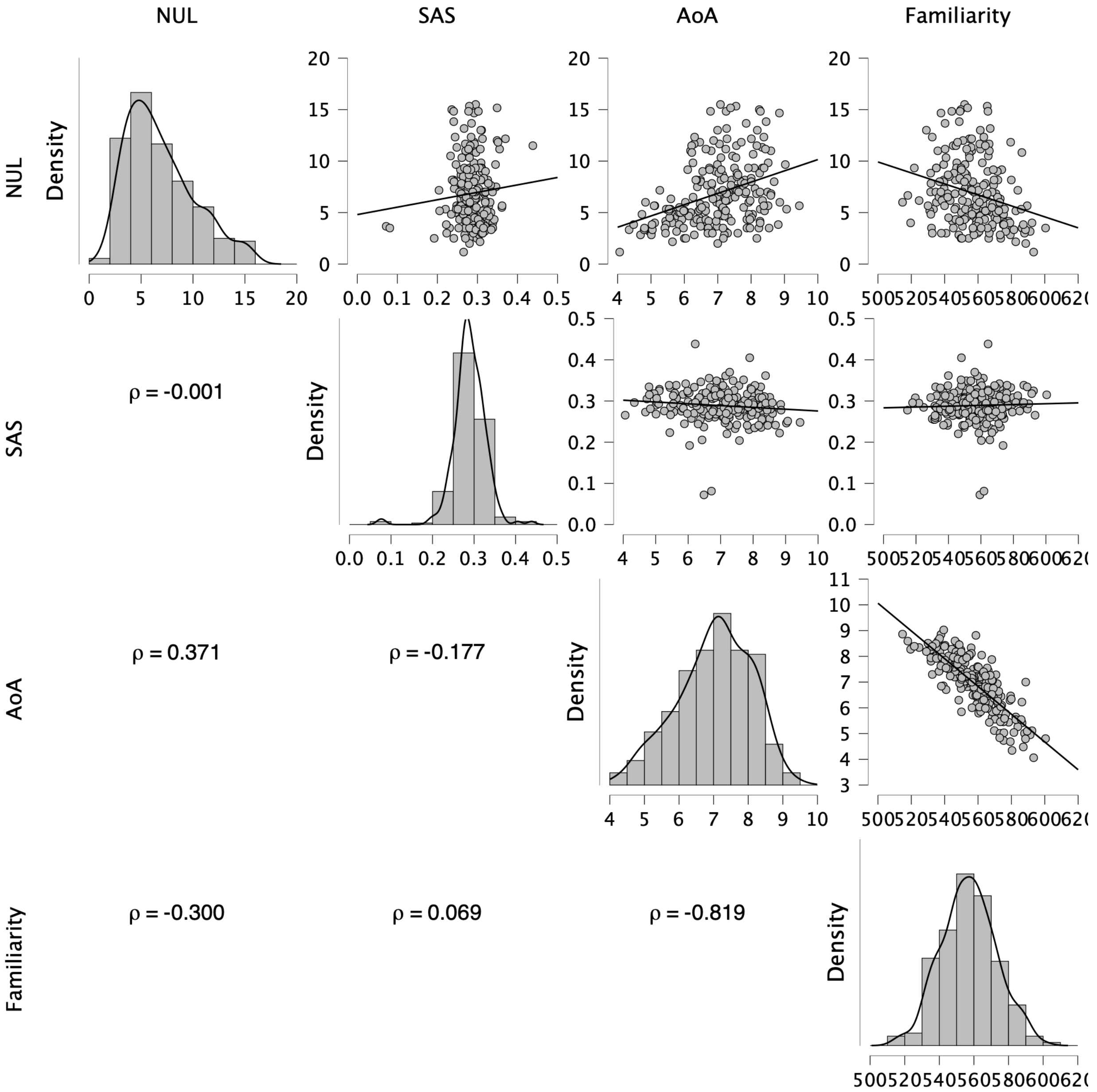

Supplementary Figure 3. **Histogram and scatter plot depicting relationship between active emotion vocabulary metrics of Study 1.** While the three metrics Number of Unique Labels (NUL), Age of Acquisition (AoA) and Familiarity are significantly correlated with each other, Semantic Agreement Score (SAS) was not correlated with any of these metrics.

Predictor	*b*	*b* 95% CI [LL, UL]	*beta*	*beta* 95% CI [LL, UL]	sr^2	sr^2 95% CI [LL, UL]	*r*	Fit
(Intercept)	7.12*	[0.29, 13.96]						
MLU	0.08	[-0.03, 0.19]	0.17	[-0.05, 0.40]	.01	[-.01, .03]	-.18**	
CEA	4.03	[-7.24, 15.30]	0.10	[-0.17, 0.37]	.00	[-.01, .01]	.32**	
NUL	0.43*	[0.06, 0.80]	0.24	[0.03, 0.44]	.02	[-.01, .06]	.27**	
SA	23.20**	[7.86, 38.54]	0.27	[0.09, 0.45]	.04	[-.01, .08]	.36**	
								R^2 = .162**<br>95% CI[.07,.24]

Note. * indicates $p < .05$. ** indicates $p < .01$.

Supplementary Table 1. **Multiple regression model with segmentation paradigm metrics predicting GERT-S scores in Study 1.** Segmentation Agreement (SA) appears as the strongest predictor of GERT-S score with the highest beta value.

Predictor	*b*	*b* 95% CI [LL, UL]	*beta*	*beta* 95% CI [LL, UL]	sr^2	sr^2 95% CI [LL, UL]	*r*	Fit
(Intercept)	5.73**	[2.72, 8.73]						
MLU	0.03	[-0.02, 0.08]	0.13	[-0.10, 0.36]	.01	[-.01, .02]	-.12	
CEA	0.69	[-4.26, 5.64]	0.04	[-0.24, 0.32]	.00	[-.00, .00]	.24**	
NUL	0.12	[-0.05, 0.28]	0.15	[-0.06, 0.36]	.01	[-.01, .03]	.18**	
SA	10.64**	[3.90, 17.38]	0.29	[0.11, 0.48]	.04	[-.01, .09]	.33**	
								R^2 = .117**<br>95% CI[.04,.19]

Note. * indicates $p < .05$. ** indicates $p < .01$.

Supplementary Table 2. **Multiple regression model with segmentation paradigm metrics predicting STEU-B scores.** While the overall model is significant and the individual zero-order correlations between STEU-B and CEA, SA and NUL are significant, only SA incrementally predicts STEU-B scores when controlling for the other metrics.

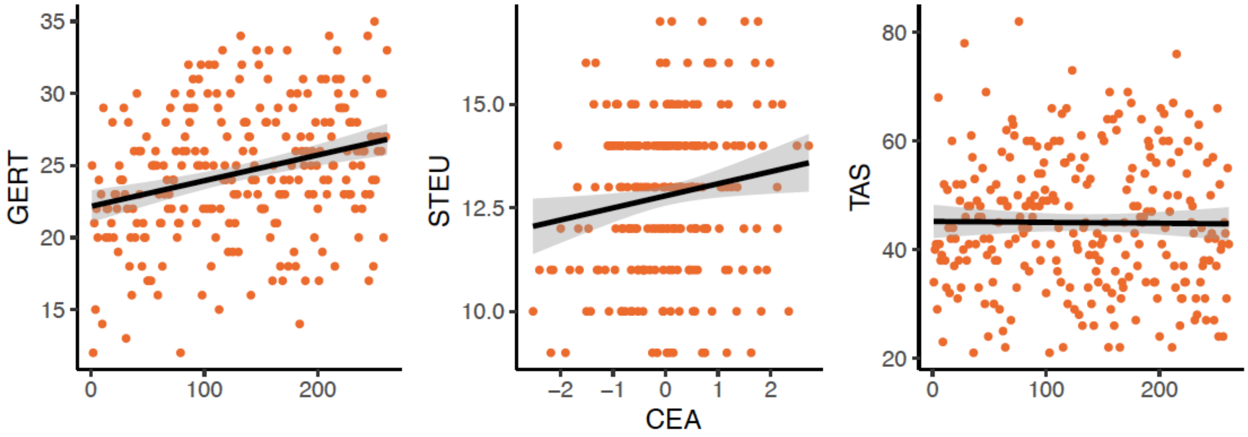

Supplementary Figure 4. **Study 2 convergent validity of CEA.** CEA was positively correlated with both GERT-S and STEU-B scores, but was not correlated with TAS-20 scores.

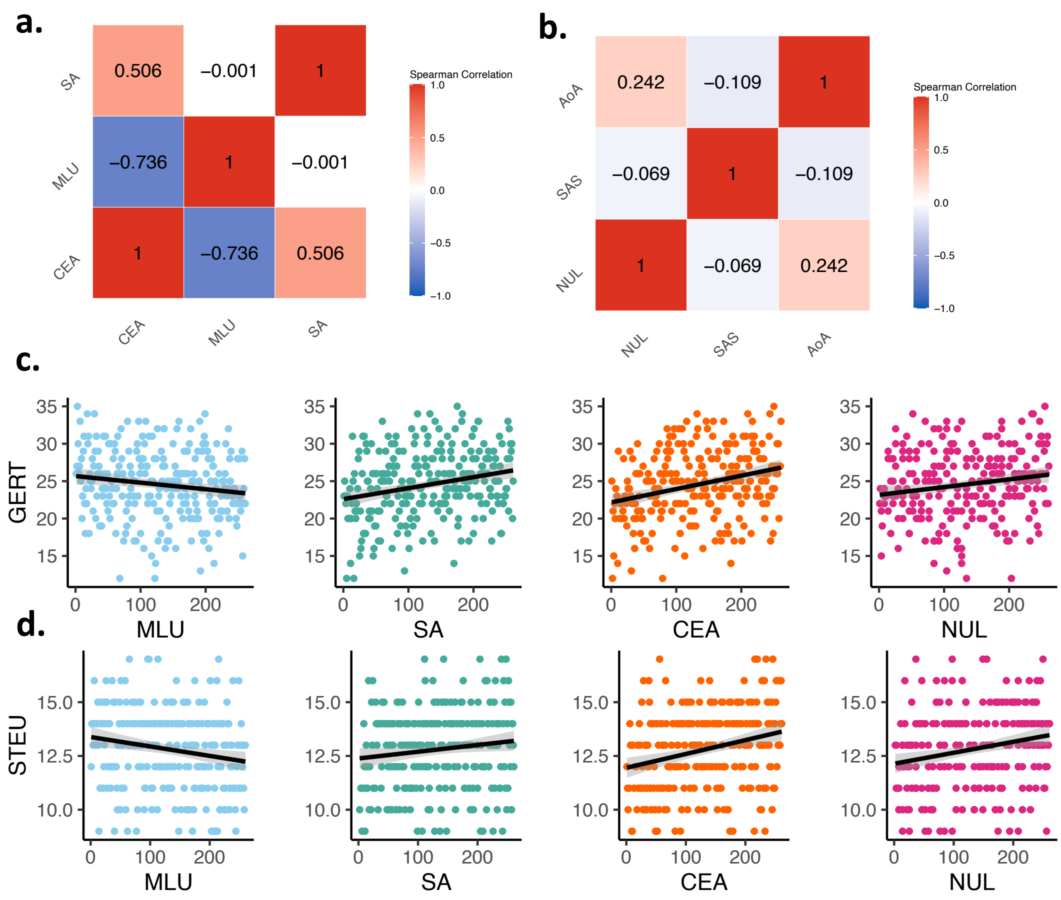

Supplementary Figure 5. **Correlation within metrics and between metrics and established measures in Study 2. a**, While the emotion segmentation metric Consensus Event Agreement (CEA) significantly correlated to both Mean Length of Unit (MLU) and Segmentation Agreement (SA), the latter two metrics did not correlate with each other. **b,** While the two active emotion vocabulary metrics Number of Unique Labels (NUL) and Age of Acquisition (AoA) significantly correlated with each other, neither correlated with Semantic Agreement Score (SAS). **c,d,** While three paradigm metrics (SA, CEA, NUL) correlated with both GERT-S scores and STEU-B scores in the expected direction, NUL was not correlated with either. The graph is the scatterplot of ranked data.

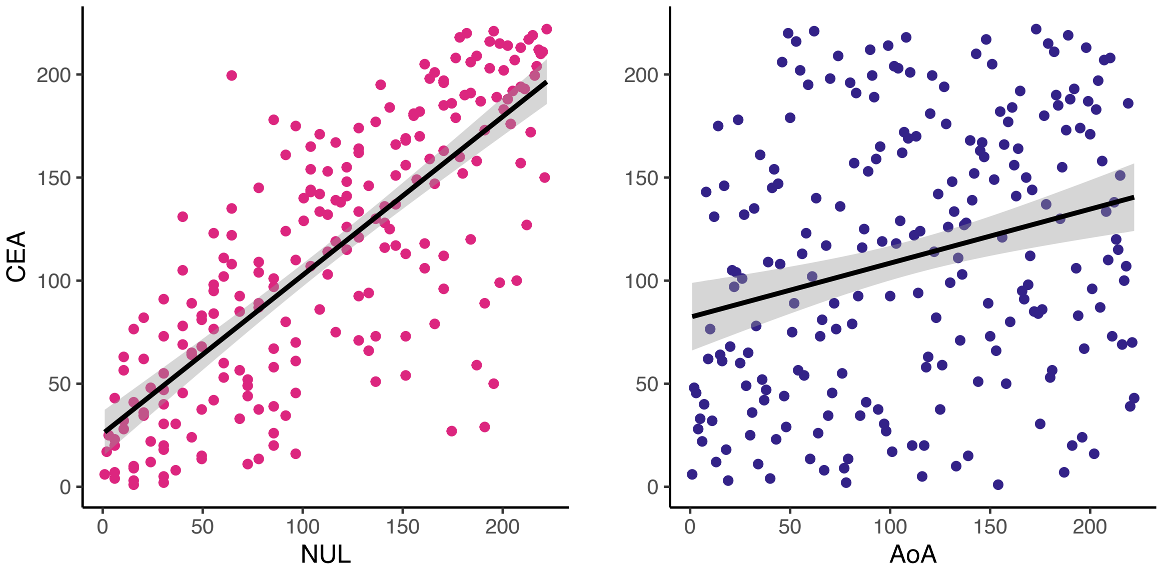

Supplementary Figure 6. **Correlation between emotion segmentation metric and active emotion vocabulary metric in Study 1.** The primary emotion segmentation metric Consensus Event Agreement (CEA) is positively correlated with both active emotion vocabulary metrics, Number of Unique Labels (NUL) and Age of Acquisition (AoA) (NUL, AoA), suggesting that people with better segmentation performance also possess larger and more advanced emotion vocabularies. The effect was replicated in Study 2.

Supplementary Note 3. Semantic Agreement Score (SAS) and Semantic Distinctiveness Score (SDS) calculation and analysis

Overview. To quantify the agreement between the sentiment expressed by the participant's label semantics and the average sentiment of the group, we calculated a Sentiment Agreement Score (SAS). For each label provided by a participant, we quantified meaning by examining the highest loading on a set of emotion categories (13 or 43 across two metrics calculated) within the AffectVec[1] word embedding space. We then compared the similarity of participants' label loadings to that of the group average to quantify semantic agreement. Details of this calculation are provided below.

While SAS aims to capture the degree of semantic agreement of the individual's labels with regards to the group-level semantics, we also calculated how distinctively participants represent different consensus events across emotion categories. We refer to this as the Semantic Distinctiveness Score (SDS), which captures the within-person differentiation of emotion concepts across trials. Here we focus on a more narrow set of 6 emotion categories, based on prior research demonstrating meaningful individual level variation in the ability to distinguish between this more limited set of emotion categories[2,3].

Semantic Agreement Score (SAS). We first compiled a data file containing emotion labels generated by participants for all consensus events, as well as the consensus event timestamp and participant ID corresponding to each label. We expanded the stopword list in the tm package by including non-sentiment labels, and removed the expanded stopwords, extra blank spaces, numbers, and punctuations from the emotion labels in the file. We then located each label of the preprocessed body of labels in the list of target words of the emotion database AffectVec[1]. AffectVec provides scores that reflect relatedness between over 70000 target words and about

200 different emotion categories, and correlates strongly with human judgements compared with other word embedding resources. For each label generated by the participants we either found its exact matching target word in AffectVec, or, in the case when there was no exact same match, the target word that is synonymous or shared the semantic root with the label (e.g., “hope” as the target word of the label “hopeful”). Labels with typos were manually corrected and matched accordingly. We then identified, among the 13 discrete emotions categories (amusement, anger, awe, contempt, disgust, embarrassment, fear, happiness, interest, pride, sadness, shame, surprise), the emotion with the highest loading score with the target word. These emotion categories were focused on given the clear predictions in the literature regarding the non-verbal behavior that is associated with each category and in line with prior work examining emotion inferences from complex stimuli[4]. The highest loading out of the 13 emotion categories was used to determine the category and loading for a given label. In other words, each label generated by participants eventually corresponded to a loading score on one of the 13 discrete emotions, via intermediate matching with target words in the database. This semantic consensus approach also includes non-emotion labels generated by participants. For instance, if a participant generates the word “rant,” based on the AffectVec embedding, the word will have the highest loading score on the emotion category of anger (0.4049), among other categories. The word will hence be considered in consensus with words that directly refer to anger (e.g., “angry,” which loads on anger at 0.7573).

For each consensus event, we summed up the semantic loading of emotion labels across individuals on each basic emotion, resulting in the semantic structure of the said consensus event in the form of a 1*13 matrix. Take the example that five participants identified consensus event *A* and generated a total of 7 emotion labels. Two labels had semantic loadings on the basic

emotion “fear” (i.e., their target words had the highest association with the basic emotion “fear”) while the remaining five had semantic loading on the basic emotion “sad.” We then summed up the semantic loadings of the two labels and stored them under the emotion “fear,” repeated the same process with the other five labels and stored them under the emotion “sad.” Consensus event *A* hence would have a semantic structure of a 1*13 matrix with values reflecting semantic loading under “sad” and “fear.” The aggregation of the semantic structures of all consensus events resulted in the group-level semantic structure, which was an n*13 matrix (n = number of consensus events).

We repeated the same procedure to generate the semantic structure for each participant. Take the example of a participant identified 2 out of n consensus events and generated a total of 3 emotion labels. Among the 3 labels, one corresponded to consensus event A with semantic loading on “anger” and 2 for consensus event B with semantic loading on “surprise” and “pride.” The participant would hence have a semantic structure of 2*13 matrix, with loadings for the three emotions and the two consensus events identified. If two labels of the same consensus event had semantic loadings on the same emotion, the resulting value corresponding to that basic emotion would be the sum of the two semantic loadings.

To compute the Semantic Agreement Score of a participant, we first calculated the Spearman correlation coefficient between the group-level semantic structure and the semantic structure of the said participant, resulting in a 1*13 matrix of correlation scores. We then averaged across the correlation scores of *identified* consensus events, resulting in the final single SAS score. As described, we quantified this agreement by evaluating the semantic relatedness of participants’ labels with 13 discrete emotion categories (referred to as SAS13).

In Study 1, we computed the internal reliability of the SAS13 metric by averaging the spearman correlation across the consensus events of each video. Results indicated low reliability of SAS13 (ω_T = 0.42). Correlation with the primary emotion segmentation metric CEA also yielded insignificant results (Spearman's ρ = -.067; 95% CI, (-0.20, 0.07), P = .858). Given these result, further exploratory analyses were conducted to address the low reliability and robustness of SAS13. First, we developed an alternative construction of the group-level semantic structure. The original computation used the sum of the semantic loadings of all labels generated during a given consensus event (CE). Instances where one participant generated multiple labels loading onto one emotion for a given CE, would inflate the loading score of that emotion. To address this concern, we recomputed by using the loading average for a given emotion of a given CE across the number of labels loading onto that emotion for that CE. For each CE, we averaged the sum of loadings onto each emotion for each CE by the number of labels generated at each CE that were associated with each emotion. To account for the varied identifiability of each CE, we further averaged the loading distribution of each CE over the total number of people identified each CE. We then correlated this alternative group-level semantic structure with the individual semantic structure, calculated the same as with the original SAS13. The alternative computation still resulted in low reliability (ω_T = 0.34) and insignificant correlation with CEA (ρ = -.102; 95% CI, (-0.23, 0.03), P = .947).

The results above suggest that the issue might not reside in the construction of semantic structure at the level of each consensus event. We hence preserve the computation of group-level semantic structure using sum loadings and explore the alternative computation using instead a wider range of emotions. We calculated an additional version of SAS score with respect to a total of 43 discrete emotion categories (referred to as SAS43), capturing a wider range of emotions

contained in the labels (see Supplementary Fig.6). This set of 43 categories was based on previous literature studying categorization of emotion in various forms of stimuli (video, prosody, music, etc.)[5–9]. Compilation and removal of repetitions of these categories across different stimuli types resulted in 47 discrete categories. Among these categories, 33 were directly included within the 200 emotional words provided by AffectVec. The unmatched 14 categories were included within the target words of AffectVec and were replaced with its highest correlated emotional word in the database. Final category list contained 43 discrete emotions categories that capture more nuanced subjective emotional experience elicited by movie clips in the current study (see Supplementary Figure.6). Both SAS13 and SAS43 follow the same calculation process as detailed above. We then computed the internal reliability of SAS43 and its correlation with CEA. Results showed that expanding the range of discrete emotions indeed increased the internal reliability of SAS43 (ω_T = 0.59) and its correlation with CEA (ρ = 0.092; 95% CI, (-0.04, 0.22), P = .073). We further tested whether, instead of using the highest loaded emotion as the single proxy for the semantic of the label, using the loading distribution of all 43 emotions would improve the internal reliability of SAS. We repeated the computation with this new loading scheme while the loading sum as group-level semantic structure. The results did not suggest higher internal reliability (ω_T = 0.27) or greater correlation with CEA (ρ= -0.054; 95% CI, (-0.18, 0.08); P = .801).

The final computation method with the greatest internal reliability and correlation with CEA (i.e., using 1) 43 discrete emotion categories; 2) group-level semantic structure based on loading sum; 3) single highest loaded emotion and its corresponding loading score for each label) is hence propagated to Study 2, as well as test-retest reliability using Study 2 stimuli. Replicating Study 1, the SAS showed adequate internal reliability (ω_T = 0.50) though its correlation with

CEA remained nonsignificant ($\rho = 0.023$; 95% CI, (-0.11, 0.15), $P = .356$). However, the test-retest reliability of SAS was rather weak (ICC = .336; $P < .001$; $r = .344$; 95% CI, (0.19, 1.00), $P < .001$). We hypothesized that this low test-retest reliability might be attributed to the nuances lost as the data was collapsed into one single score. To test our hypothesis, we first correlated the semantic structures of the labels generated by each individual at test and retest, resulting in the median correlation of 0.463. This result suggests that the semantic structure of the labels generated by the participants were not that consistent across two weeks. We further correlated the Spearman correlation matrices (calculated by correlating the group-level semantic structure with the individual semantic structure) for each individual across two weeks, which similarly yielded low consistency as the median correlation was 0.405. These results suggest that the low test-retest reliability might be mainly due to the insufficient sensitivity of AffectVec loading in distinguishing between high and low levels of agreement with the group semantic structure. In AffectVec dataset, labels that are of the same semantics yet different linguistic forms may load differently on the same emotion. For instance, while the label “anxiety” loads on the emotion “fear” with the score of 0.7502, the label “anxious” only loads with the score of 0.4725. On the other hand, synonyms such as “concern” and “worry” may also have varied loading on the same emotion “fear” (concern loads at 0.5324, while worry loads at 0.7664). These variances may render people who produce similar labels for the same consensus event across two weeks attain distinct SAS due to the varied loading scores.

Semantic Distinctiveness Score (SDS). Similar to SAS, to calculate semantic distinctiveness scores, we constructed individual-level structures of semantic loadings onto 6 discrete emotion categories (i.e., fear, surprise, happiness, anger, disgust, sadness) for each of the consensus events a participant identified. The semantic loadings were based on the labels

generated during emotion segmentation of each consensus event using the AffectVec loadings for these specific emotion categories. For each participant, we first located the labels generated at each consensus event in AffectVec and compiled the corresponding semantic loadings on all 6 discrete emotions. If more than one label was provided for the event by a participant, then loadings were averaged to capture the general semantic representation for the single consensus event. This yielded a $n * 6$ matrix for each subject, where n equals the number of critical events identified by the participant in the emotion segmentation task. We then calculated the squared Euclidean distance between the 6 n-length vectors of semantic loadings, resulting in a distinctiveness score between each pair of emotion categories (i.e., Euclidean distance between semantic loading matrices)[2]. The lower the distinctiveness score, the more semantically similar the labels provided for consensus events were with respect to the two emotion categories.

To capture the within- and between-valence variation in semantic structures, we then computed three different scores. To capture the semantic distinctiveness between each within-valence (i.e., angry, disgust, sad and fear) pair of negative emotions, we averaged the pairwise distinctiveness score between each of these negative emotions. We refer to this score as the SDS negative-negative (for short *SDS_NN*). To capture the cross-valence distinctiveness in semantics, we calculated the average of the distinctiveness score for happy-sad, happy-anger, happy-fear and happy-disgust pairs. We refer to this score as SDS positive-negative (for short *SDS_PN*). Finally, to capture distinctiveness in semantics between an ambiguously valenced emotion (surprise) and the other negative emotions, we calculated the average of the distinctiveness score for surprise-sad, surprise-anger, surprise-fear and surprise-disgust pairs. We refer to this score as SDS ambiguous-negative (for short *SDS_AN*).

To investigate the relationship between the semantic distinctiveness score and emotion segmentation, we also conducted the Spearman correlation test between each of the 3 SDS metrics with CEA in Study 2 (*N*=252). All three SDS metrics derived from the semantic loading of the labels were significantly correlated with CEA (SDS_NN: ρ = .759; 95% CI, (0.70, 0.81), *P* < .001; SDS_PN: ρ = .774; 95% CI, (0.72, 0.83), *P* < .001; SDS_AN: ρ = .847; 95% CI, (0.81, 0.88), *P* < .001). The same pattern was replicated in Study 2 (SDS_NN: ρ = .724; 95% CI, (0.66, 0.79), *P* < .001; SDS_PN: ρ = .760; 95% CI, (0.70, 0.82), *P* < .001; SDS_AN: ρ = .832; 95% CI, (0.79, 0.87), *P* < .001). These results suggest that the conceptual structure similarity measured using people's own active emotion vocabularies (i.e., label generated during emotion segmentation tasks) is associated with people's emotion segmentation performance.

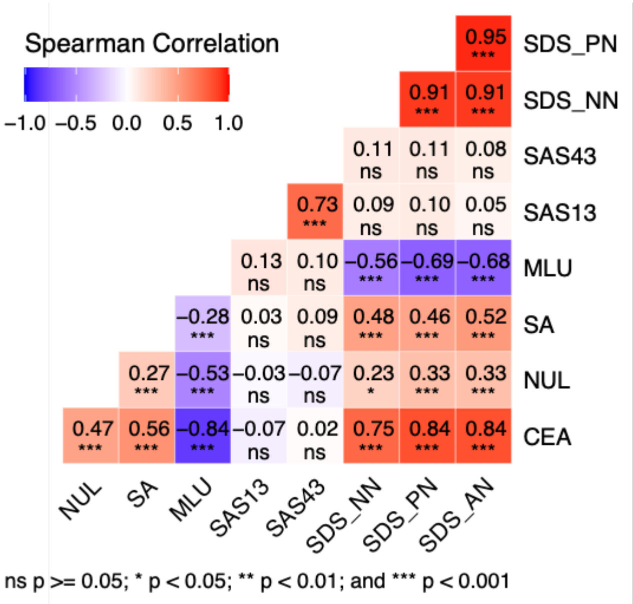

Supplementary Figure 5. **Pairwise correlation between all paradigm metrics in Study 1 (MLU, SA, CEA, NUL, SAS, SAS2)**.

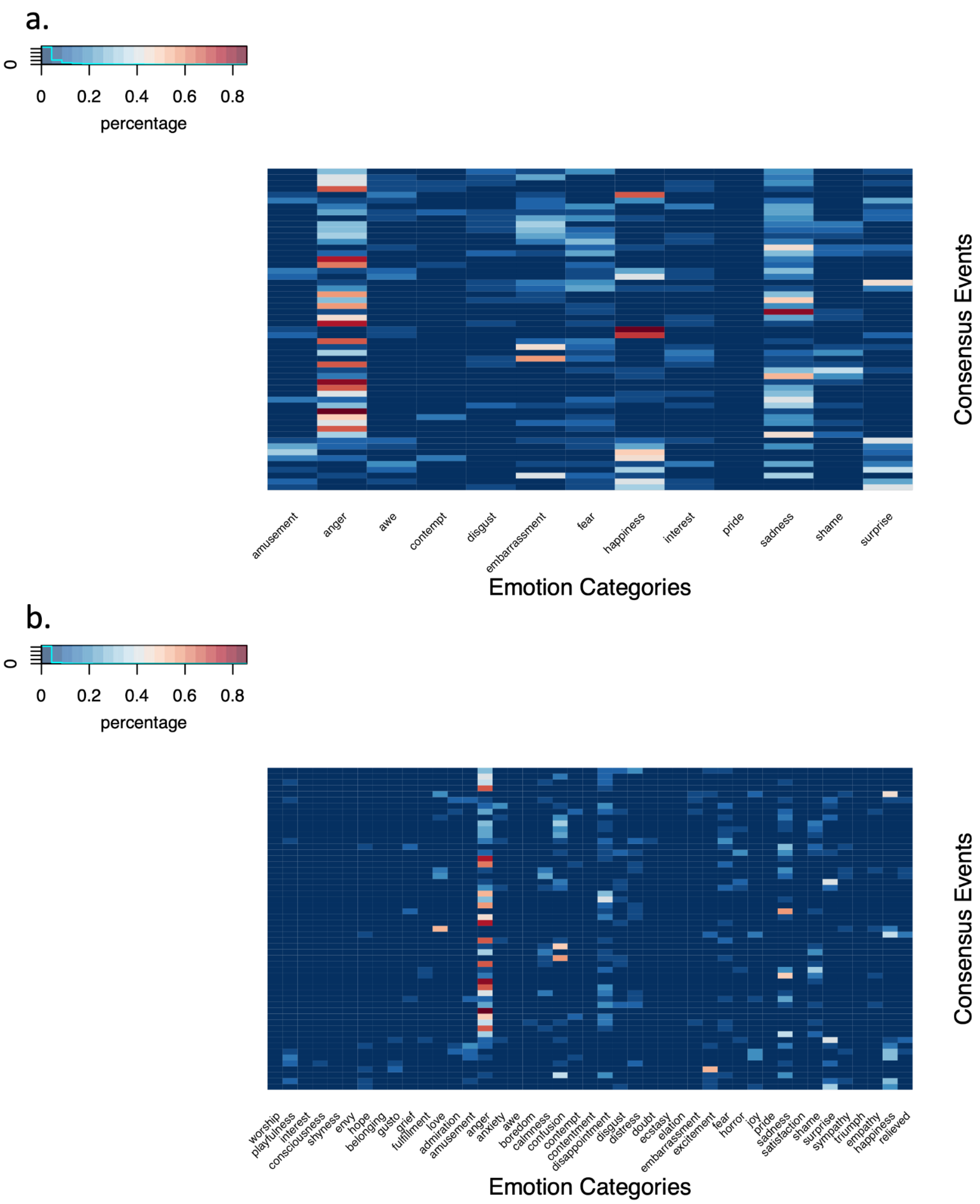

Supplementary Figure 6. **Semantic complexity with 13 versus 43 discrete emotion categories.** Compared to the version with 13 discrete emotion categories, additional emotions such as contentment and confusion are capturing additional meaningful variation in the video stimuli.

Supplementary Note 4: Additional analysis with Psychological Well-Being Scale (PWB) and Autism Spectrum Quotient - 10 items (AQ-10)

In Study 1 and 2 we used the Positive Relations with Others subscale of the Psychological Well-Being Scale (PWB) to examine the concurrent validity of the paradigm metrics (i.e., MLU, SA, CEA, NUL; SAS was excluded for its insignificant correlations with other paradigm metrics). We expected that dynamic emotion perception should be associated with positive social relationships[10], reflected as the correlation between the Emotion Segmentation Paradigm metrics and the Positive Relations with Others subscale scores. We conducted pairwise Spearman correlation (Bonferroni corrected for multiple comparisons) between the four paradigm metrics and the subscale scores. The results in Study 1 suggested that Positive Relations with Others subscale scores was not associated with any of the emotion segmentation metrics (MLU: ρ = -.102; 95% CI, (-0.23, 0.04), P = .268; SA: ρ = .035; 95% CI, (-0.01, 0.17), P = 1.00; CEA: ρ = .087; 95% CI, (-0.050, 0.22) P = .398) but was positively associated with the active emotion vocabulary metric (ρ = .127; 95% CI, (-0.01, 0.26), P = .031). The lack of association was replicated in Study 2 (MLU: ρ = .001; 95% CI, (-0.12, 0.13), P = 1.000; SA: ρ = -.133; 95% CI, (-0.26, -0.01), P = 1.000; CEA: ρ = -.04; 95% CI, (-0.17, 0.08), P =1.000), where the association with active emotion vocabulary metric was no longer present (NUL: ρ = .059; 95% CI, (-0.06, 0.18), P = .696). These results are partially consistent with the findings that larger positive active emotion vocabularies are associated with higher psychological well-being[11], yet also allude that the link between psychological well-being and dynamic emotion perception ability may be less robust. There are caveats that might explain this latter lack of association. On the one hand, such global and retrospective evaluations of subjective well-being (or relations with others) may diverge from the actual experience[12]. Furthermore, in

the case of relations with others, verification from these interaction partners is also absent, leaving possibility for deviation between subjectively perceived and actual relationship quality.

In Study 1, to further test for the robustness of the metrics in a nonclinical sample, we used AQ-10 scores as the screening tool to distinguish the subsample that was considered as non-autistic (i.e., scoring less than 6). We repeated the main analysis with the non-autistic subsample, including tests for the reliability of paradigm metrics, convergent validity of the metrics against GERT-S and STEU-B scores, and focal analysis on the association between CEA and two active emotion vocabulary metrics (NUL & AoA). The results largely replicated that produced with the full sample. Most of the main metrics, namely MLU (ω_T = 0.9 for affective stimuli, 0.74 for non-affective stimuli), CEA (ω_T = 0.86 for affective stimuli, 0.79 for non-affective stimuli), and NUL (ω_T = 0.95 for affective stimuli) were still highly reliable. SA, on the other hand, demonstrated medium level reliability (ω_T = 0.65 for affective stimuli, 0.58 for non-affective stimuli).

In terms of convergent validity of the non-autistic sample metrics, SA, CEA and NUL still positively correlated with the GERT-S score (SA: ρ = .276; 95% CI, (0.14, 0.42); P < .001; CEA: ρ = .298; 95% CI, (0.16, 0.42); P < .001; NUL: ρ = .252; 95% CI, (0.11, 0.38); P = .001) and the STEU-B score (SA: ρ = .289; 95% CI, (0.16, 0.42); P < .001; CEA: ρ = .272; 95% CI, (0.14, 0.40); P < .001; NUL: ρ = .194; 95% CI, (0.06, 0.34); P = .019). MLU still correlated with GERT-S score (ρ = -.181; 95% CI, (-0.31, -0.04); P = .03), but not with the STEU-B score (ρ = -.106; 95% CI, (-0.25, 0.03); P = .368). The other active emotion vocabulary metrics SAS correlates with GERT-S (ρ = .179; 95% CI, (0.04, 0.32); P = 0.03) but not STEU-B scores (ρ = .11; 95% CI, (-0.01, 0.26); P = 0.292).

We also repeated the pairwise analysis between the emotion segmentation metrics and the active emotion vocabulary metrics of Study 1 (Bonferroni corrected for multiple tests).

Replicating the results with the full sample, each of the three emotion segmentation metrics still significantly correlated with both NUL (MLU: ρ = -.856; 95% CI, (-0.91, -0.78); $P < .001$; CEA: ρ = .738; 95% CI, (0.66, 0.81); $P < .001$; SA: ρ = .415; 95% CI, (0.28, 0.54); $P < .001$) and AoA (MLU: ρ = -.227; 95% CI, (-0.36, -0.09); P = .005; CEA: ρ = .240; 95% CI, (0.11, 0.37); P = 0.002; SA: ρ = .217; 95% CI, (0.08, 0.36); P = 0.007). These results suggest that within the non-autistic sample, people with better segmentation performance that segment at a more fine-grained manner also produced more distinct emotion labels that were acquired at an older age and arguably more complex and advanced.

Supplementary Note 5: Representational Similarity Analysis (RSA) task and analysis

Representational similarity analysis (RSA) captures individual variance in representing emotional concepts. We invited 167 participants with good effort (i.e., have segmented all 9 videos in the emotion segmentation task) from Study 1 to participate in the RSA task. The task consisted of 40 emotional features that were directly selected from materials used by Brooks and Freeman[2]. These features included frequently used terms representing bodily reactions (e.g. heart-racing), feelings (e.g., loving) and actions (e.g., crying) related to 6 basic emotion categories (i.e., fear, surprise, happiness, anger, disgust, sadness). Participants rated the relatedness of the emotional features with respect to each of the 6 basic emotion categories on a 7-point Likert scale (1= not at all, 7=extremely).

For each participant, we constructed six 1*40 matrices by compiling the ratings of 40 emotional features for the 6 emotion categories. We then calculated the squared Euclidean distance between the 6 matrices of feature ratings, resulting in a similarity score for each emotion category (i.e., Euclidean distance between feature matrices) with respect to the other emotion categories. The lower the score, the more conceptually similar the two emotions were.

To capture the within- and between-valence variation in the conceptual similarity structures, we then averaged the similarity scores with respect to each basic emotion category across the 4 negative emotion categories (i.e., angry, disgust, sad and fear). This resulted in 6 mean similarity scores termed *meanNeg*, each score representing each emotion category's conceptual similarity with the negative emotion categories on average.

Within these 6 *meanNeg* scores, we averaged the 4 scores corresponding to the 4 negative basic emotion categories, resulting in a single score termed *neg_neg* representing the conceptual similarity structure *within* the negative valence. For the two remaining *meanNeg* scores, the score

corresponding to the basic emotion category "happiness" was termed *pos_neg*, representing the between-valence conceptual similarity between positive and negative emotions. The score corresponding to the basic emotion category "surprise" was termed *amb_neg*, representing the between-valence conceptual similarity between emotions with ambiguous valence and negative emotions.

We conducted Spearman correlation analysis (Bonferroni corrected for multiple comparison) between the primary emotion segmentation metric CEA and each of the three RSA metrics. As some of the participants invited for the RSA task were low-effort individuals or did not complete both sessions of the study, the correlation sample size consisted of only 144 subjects. Results showed that none of the three metrics correlated with CEA (neg_neg: Spearman's ρ = .0735; 95% CI, (-0.084, 0.238), P = .569; pos_neg: ρ = .136; 95% CI, (-0.03, 0.299), P = .157; amb_neg: ρ = .0313; 95% CI, (-0.140, 0.202), P = 1.00). The result suggests that the emotion segmentation performance did not appear to be associated with within-valence and between-valence emotion conceptual dissimilarity.

Supplementary Note 6. Exploratory analysis using Signal Detection Theory

Overview. The paradigm metrics focusing on dynamic emotion perception, namely the Mean Length of Unit (MLU), Segmentation Agreement (SA) and Consensus Event Agreement (CEA), aim to capture individual differences in participants' emotion segmentation behaviors. However, variance in these metrics may be driven by individual differences in 1) participants' sensitivity at discriminating between emotion and non-emotion events and 2) participants' criterion that justifies segmentation behaviors. For instance, people may pause less frequently (i.e., have higher MLU) either because they are not sensitive to detect emotion events or because they have a more stringent threshold for segmenting emotion events. To unpack performance, we adapted measures from the literature of signal detection theory literature. We computed two metrics, d' and c, that respectively represent individual differences in sensitivity and criterion. A greater value of d' suggests greater sensitivity at discriminating instances of emotion from non-emotion, while greater value of c suggests a more conservative threshold for the identification of an emotion event.

SDT metrics computation. Signal detection theory in the field of psychology is usually applied to quantify discrimination between the perception of signal (stimuli) and noise (no stimuli)[13]. In the current study, we treated each consensus event window as 'signal trials', and intervals between consensus events as 'noise trials' (we also treated the beginning and end of the video as noise trials). Across all videos, this resulted in 55 signal trials and 56 noise trials for emotion trials and 20 signal trials and 22 noise trials for control trials in Study 1 (N=222), and 60 signal trials and 68 noise trials in Study 2 (N=261). Participants responses were coded as the following: Pauses within each signal trial were coded as a "hit;" failures to pause within each

signal trial were coded as a “miss;” pauses within each noise trial were coded as a “false alarm;” and failures to pause within each noise trial were coded as a “correct rejection.”

For each study, we computed two SDT metrics, *d’* and *c*. We first computed the hit rate (*hr*), calculated as the sum of number of hits and misses divided by number of hits, and the false alarm rate (*far*), calculated as the sum of number of false alarms and correct rejections divided by number of false alarms. We then calculated *d’*, the metric represented sensitivity, as the difference between the z-score of hit rate (*zhr*) and the z-score of false alarm rate (*zfar*), and *c*, the metric representing criterion, as half the negative sum of *zhr* and *zfar*[14,15]. We computed SDT metrics in two ways: across all videos (our primary approach) or within each video and then averaged the SDT parameters across the videos (which we treat as an alternative method, primarily for assessing reliability, *d’_alt* and *c_alt* henceforth). Some participants did not have any hits or false alarms within certain videos, so we adopted loglinear transformation to address these extreme proportions[16]. We first computed the proportion of signal trials and noise trials for each video, then added the former value to the number of hits and the latter value to the number of false alarms. We perform this transformation on all participants’ entries for this alternative measure.

Reliability of SDT metrics. To test the internal reliability of the SDT metrics, we computed the Omega total scores[17] for *d’* and *c* for each video (corrected via loglinear transformation). In the Study 1 sample, *d’* did not evidence strong reliability ($\omega_T = 0.46$ for affective stimuli, $\omega_T = 0.38$ non-affective stimuli) while *c* did ($\omega_T = 0.92$ for affective stimuli, $\omega_T = 0.85$ non-affective stimuli). These results were replicated in the Study 2 sample for both *d’* ($\omega_T = 0.57$) and *c* ($\omega_T = 0.88$). The issue with low reliability of *d’* was also encountered by Karmon-Presser and colleagues[18] (2018), who calculated *d’* as representation of the ability to

differentiate between external stimuli as evidence for subjective emotion experience. They attributed issue to its computational approach, as psychometric theory suggests that reliability tends to increase with a combination of measures[18,19]and measures based on difference tend to be unreliable[20]. As *d'* is calculated based on subtraction (*zhr*-*zfar*), it may therefore have lower reliability than *c*, which is calculated based on summation (-0.5(zhr+zfa)). It is possible that these same challenges are present in our use of d' as an individual difference measure.

Convergent validity of SDT metrics. We evaluated the convergent validity of the two SDT metrics by computing the pairwise correlations (Bonferroni corrected for multiple comparisons) between each metric and each of the two convergent validity scales, namely the Geneva Emotion Recognition Test (GERT-S) and Situational Test of Emotion Understanding-Brief (STEU-B) (Supplementary Table 3). We evaluated the validity for both methods of computation. In Study 1 sample (emotion trials only), *d'* (calculated across all trials) correlated positively and more strongly with GERT-S and STEU-B scores than *d'_alt* (averaged SDT metrics which were calculated at the level of each video). While the positive correlations between *d'* and both scale scores replicated with Study 2 sample, *d'alt* did not significantly correlate with STEU-B scores ($P = 0.10$ after correction). The two *d'* variants also positively correlated with each other in both samples (Study 1 sample: $\rho = .945$, 95% CI [0.924, 0.964], $P < .001$; Study 2 sample: $\rho = .956$, 95% CI [0.939, 0.970], $P < .001$). Similarly, *c* and *c_alt* positively correlated with each other in both samples (Study 1: $\rho = .993$, 95% CI [0.992, 0.996], $P < .001$; Study 2: $\rho = .990$, 95% CI [0.988, 0.994], $P < .001$). Both variants of *c* negatively correlated with both GERT-S and STEU-B scores, with *c* correlated more strongly than *c_alt*. Given the results, we decided to use *d'* and the corresponding *c* computed across all trials (rather than at the video level) for further analysis because they demonstrated 1) greater effect sizes for

convergent validity, 2) strong correlations with the alternative computations, and 3) are computed based on a higher number of trials, which may help to mitigate the unreliability observed for the video level *d'* metric. To further probe the unique relationships between *d'* and *c* and our measures of convergent validity, we conducted an exploratory multiple regression analysis and set up separate models with GERT-S and STEU-B scores as outcome variables. For both samples, when controlling for the other variable, *d'* and *c*, both still predicted GERT-S and STEU-B scores (in the same directions we observed in the pairwise correlations) (Supplementary Table 4-7). These results collectively suggest that better performance in an established standardized emotion perception task and an individual's situational knowledge about emotions were both associated with greater sensitivity at discriminating emotion events among relevant contextual information, but also with more liberal threshold at identifying the emotion events.

Relationship with emotion segmentation metrics. Prior literature suggests that, in the presence of certain response biases required by the environment, people with low sensitivity may maximize the expected value of their decision by adopting a more liberal criterion than those with high sensitivity[21]. In the Emotion Segmentation Paradigm, the instruction (i.e., to pause the video as many times as needed) may have motivated people to segment as much as possible instead of refraining from segmentation, hence encouraging a response bias towards segmenting more liberally. We therefore predicted that given this general bias, those with lower sensitivity may adopt a more liberal criterion. Study 1 sample, however, contradicted this prediction, such that we observed a negative correlation between *d'* and *c* (ρ = -.280, 95% CI [-0.410, -0.151], P < .001), suggesting that a more liberal criterion is associated with greater sensitivity. This might be due to the largely negative *d'* (range = -1.77-0.65, *Mean* = -.49, *SD* =.37) and largely positive *c* (range = -0.67-1.68, *Mean* =.51, *SD* = .40). The correlation was not replicated in Study 2

sample (ρ = -.099, 95% CI [-0.223, 0.028], *P* = .056), which had a more widely distributed *d’* (range = -2.58-2.15, *Mean* = -.32, *SD* =.80) and *c* (range= -1.07-1.45, *Mean* = .32, *SD* =.41), indicating that the relationship between these two parameters is not stable across samples.

For both samples, we further investigated the relationship between SDT metrics and the main metrics of the study, namely the emotion segmentation metrics (Mean Length of Unit, Segmentation Agreement, Consensus Event Agreement) and the active emotion vocabulary metrics (Number of Unique Labels, Age of Acquisition of the labels) by computing the pairwise correlations (Bonferroni corrected for multiple comparisons) between each of the SDT metrics and 1) each of the emotion segmentation metrics (Supplementary Table 8) and 2) each of the active emotion vocabulary metrics (Supplementary Table 9). Both *d’* and *c* correlated with SA and CEA in the expected directions for both samples. That is, *d’* positive correlated with both SA and CEA suggesting that these metrics partially reflect greater ability to discriminate consensus emotion events from non-events. On the other hand, *c* correlated negatively with both metrics, suggesting that having a more liberal threshold (i.e., a lower value of *c*) is associated with stronger segmentation performance.

We also found that *c* was positively correlated with MLU. This suggests that segmenting with a fine grain (as indicated by a low MLU) is related to having a liberal threshold (as indicate by a low *c*). In contrast, we did not see that *d’* was related to MLU, suggesting that segmenting with a fine grain is not related to the ability to discriminate group consensus emotion events from non-events.

Relationship with active emotion vocabulary metrics. We next examined the relationships between our SDT parameters and active emotion vocabulary metrics (Number of Unique Labels, Age of Acquisition of the labels) (Table 7). Consistent with the links between *c*

and the original segmentation metrics (CEA, SA), we observed that *c* was significantly negatively correlated with both active emotion vocabulary metrics. This indicates that lexical complexity for emotion is related to adopting a more liberal criterion to identify consensus emotion events from non-events. We found mixed evidence that *d'* related to lexical complexity. In Study 1, we observed a that there was a small but positive correlation between *d'* and the active emotion vocabulary metrics. This suggests that individuals who have more complex active emotion vocabularies were also more sensitive to discriminate consensus emotion events from non-events. This relationship was not replicated in the Study 2 sample, however.

Unpacking the link between emotion segmentation and active emotion vocabulary. The findings above suggest that the links between emotion vocabulary and segmentation performance using the original metrics (SA, CEA) could be driven by adopting a more liberal threshold at segmentation. We explore this potential explanation next by conducting exploratory multiple regression analysis and set up models with CEA (our primary emotion segmentation metric) as outcome variables. With both samples, controlling for criterion, neither active emotion vocabulary metrics continued to predict CEA (Supplementary Table 10 – Table 13). These results seem to suggest that the link between emotion segmentation and active emotion vocabulary may be mainly driven by a more liberal threshold to segment.

Integrative Data Analysis. A sensitivity analysis (a = 0.05, power = 0.8) suggests that Study 2 sample was only powered to detect an effect size as small as 0.172. As a result, we opted to conduct further analysis by combining the Study 2 sample with Study 3 sample. We constructed an integrative sample (N=381) using Study 2 sample (N=261) and Study 3 sample (N=120; using data from the first week). We then computed *d'* and *c* using the same computation method as above. Replicated previous patterns, *d'* demonstrated moderate internal consistency

(ω_T = 0.58) while *c* remained adequately reliable (ω_T = 0.88). Given that no GERT-S or STEU-B scores were collected during Study 3, we were not able to examine the convergent validity of *d'* and *c*.

The negative correlation between *d'* and *c* was replicated with the integrative sample, but the small effect size supported the instability of the relationship (ρ = -.090, 95% CI [-0.193, 0.014], *P* = .040). As with prior samples, both *d'* and *c* correlated with SA and CEA in the expected directions. As in prior analysis, only *c* was positively correlated with MLU (Table 14). These results further support the prior finding that while both the ability to discriminate group consensus emotion events from non-events (i.e., a higher value of *d'*) and a liberal threshold (i.e., a lower value of *c*) were associated with stronger segmentation performance evaluated against group consensus, only the latter is associated with a fine-grained segmentation style.

Replicating results found using the Study 2 sample, we observed a significant and strong negative correlation between *c* and both active emotion vocabulary metrics (Table 15), suggesting that there is a robust relationship between lexical complexity for emotion and a more liberal criterion to identify consensus emotion events. However, we found no significant relationship between *d'* and either active emotion vocabulary metric. This suggests that the lack of correlation between sensitivity and active emotion vocabulary metrics using Study 2 sample might not be due to low power, but rather reflects that individual's lexical complexity of active emotion vocabulary may not be associated with sensitivity at telling emotion events apart from non-emotion events.

As with prior samples, we further conducted exploratory multiple regression analysis examining whether links between emotion segmentation performance and active emotion vocabulary was driven by a more liberal criterion. We constructed separate models with CEA as

outcome predictors and each active emotion vocabulary metric, criterion, and dichotomous variable representing original sample as data source (variable named ‘SampleMarker’). Replicated previous findings, when controlling for criterion, neither active emotion vocabulary metric no longer predicted CEA (Table 16-17), adding evidence to the possibility that the link between emotion segmentation and active emotion vocabulary may be mainly driven by a more liberal threshold at segmentation.

	Study 1		Study 2	
	GERT-S	STEU-B	GERT-S	STEU-B
d'	. 265** [.14, .39]	. 265** [.14, .38]	.213** [.09, .33]	.132* [.00, .26]
d'_alt	.259** [.13, .38]	. 237** [.11, .36]	.204** [.08, .33]	.105 [-.02, .23]
c	-.260** [-.38, -.13]	-.211** [-.34, -.09]	-.209** [-.33, -.09]	-.247** [-.36, -.12]
c_alt	-.240** [-.36, -.11]	-.190** [-.32, -.06]	-.204* [-.32, -.09]	-.230** [-.35, -.11]

Note. * indicates $p < .05$. ** indicates $p < .01$.

Supplementary Table 3. **Spearman correlations between two variants of Signal Detection Theory (SDT) metrics and GERT-S and STEU-B scores with confidence intervals.** While both variants in general correlated with both GERT-S and STEU-B in the expected direction (except for *d'_alt* and STEU-B), *d'* and *c* has stronger correlation with the scale scores than the alternative computation.

Predictor	*b*	*b* 95% CI [LL, UL]	*beta*	*beta* 95% CI [LL, UL]	sr^2	sr^2 95% CI [LL, UL]	*r*	Fit
(Intercept)	26.60**	[25.26, 27.95]						
d'	4.06**	[2.04, 6.08]	0.26	[0.13, 0.39]	.06	[.00, .12]	.32**	
c	-2.57**	[-4.46, -0.67]	-0.18	[-0.31, -0.05]	.03	[-.01, .07]	-.26**	
								R^2 = .130**<br>95% CI[.05,.21]

Note. * indicates $p < .05$. ** indicates $p < .01$.

Supplementary Table 4. **Multiple Regression model with SDT metrics predicting GERT-S scores with Study 1 sample.** Both *d'* and *c* predicted GERT-S scores in the expected direction.

Predictor	*b*	*b* 95% CI [LL, UL]	*beta*	*beta* 95% CI [LL, UL]	sr^2	sr^2 95% CI [LL, UL]	*r*	Fit
(Intercept)	13.24**	[12.64, 13.83]						
d'	1.56**	[0.66, 2.46]	0.23	[0.10, 0.36]	.05	[-.01, .10]	.28**	
c	-1.01*	[-1.84, -0.19]	-0.16	[-0.30, -0.03]	.02	[-.01, .06]	-.23**	
								R^2 = .103**<br>95% CI[.04,.18]

Note. * indicates $p < .05$. ** indicates $p < .01$.

Supplementary Table 5. **Multiple Regression model with SDT metrics predicting STEU-B scores with Study 1 Sample.** Both *d'* and *c* predicted STEU-B scores in the expected direction.

Predictor	*b*	*b* 95% CI [LL, UL]	*beta*	*beta* 95% CI [LL, UL]	sr^2	sr^2 95% CI [LL, UL]	*r*	Fit
(Intercept)	26.53**	[25.61, 27.45]						
d'	2.69**	[1.26, 4.12]	0.22	[0.10, 0.34]	.05	[-.00, .10]	.24**	
c	-2.97**	[-4.61, -1.34]	-0.21	[-0.33, -0.10]	.05	[-.00, .09]	-.23**	
								R^2 = .104**<br>95% CI[.04,.17]

Note. * indicates $p < .05$. ** indicates $p < .01$.

Supplementary Table 6. **Multiple Regression model with SDT metrics predicting GERT-S scores with Study 2 Sample.** Both *d'* and *c* predicted GERT-S scores in the expected direction.

Predictor	*b*	*b* 95% CI [LL, UL]	*beta*	*beta* 95% CI [LL, UL]	sr^2	sr^2 95% CI [LL, UL]	*r*	Fit
(Intercept)	13.54**	[13.15, 13.93]						
d'	0.73*	[0.11, 1.34]	0.14	[0.02, 0.27]	.02	[-.01, .05]	.17**	
c	-1.28**	[-1.97, -0.59]	-0.23	[-0.35, -0.10]	.05	[-.00, .10]	-.24**	
								R^2 = .078**<br>95% CI[.02,.14]

Note. * indicates $p < .05$. ** indicates $p < .01$.

Supplementary Table 7. **Multiple Regression model with SDT metrics predicting STEU-B scores with Study 2 Sample.** Both *d'* and *c* predicted STEU-B scores in the expected direction.

	Study 1		Study 2	
	d'	*c*	*d'*	*c*
MLU	-. 125 [-.26, .01]	. 961** [.95, .97]	.07 [-.05, .20]	.939** [.92, .96]
SA	.723** [.66, .79]	-. 554** [-.65, -.45]	.707** [.64, .77]	-.184** [-.30, -.06]
CEA	.583** [.49, .68]	-.916** [-.94, -.89]	.520** [.42, .62]	-.872** [-.90, -.84]

Note. * indicates $p < .05$. ** indicates $p < .01$.

Supplementary Table 8. **Spearman correlations between SDT metrics and emotion segmentation metrics.** MLU was not correlated with sensitivity in either sample. Other emotion segmentation metrics correlated with both signal detection metrics in expected directions.

	Study 1		Study 2	
	d’	*c*	*d’*	*c*
NUL	.140*<br>[.01, .27]	-.862**<br>[-.91, -.80]	-.041<br>[-.17, .08]	-.868**<br>[-.91, -.82]
AoA	.141*<br>[.01, .27]	-. 262**<br>[-.39, -.14]	.015<br>[-.10, .13]	-.162**<br>[-.28, -.05]

Note. * indicates $p < .05$. ** indicates $p < .01$.

Supplementary Table 9. **Spearman correlations between SDT metrics and active emotion vocabulary metrics.** NUL correlated with sensitivity in Study 1 but not the Study 2 sample. AoA correlated with both sensitivity and criterion in both samples in the expected direction.

Predictor	*b*	*b* 95% CI [LL, UL]	*beta*	*beta* 95% CI [LL, UL]	sr^2	sr^2 95% CI [LL, UL]	*r*	Fit
(Intercept)	0.47**	[0.42, 0.51]						
c	-0.36**	[-0.40, -0.33]	-0.99	[-1.09, -0.89]	.32	[.24, .40]	-.91**	
NUL	-0.00	[-0.01, 0.00]	-0.09	[-0.19, 0.00]	.00	[-.00, .01]	.72**	
								R^2 = .834**<br>95% CI[.80,.86]

Note. * indicates $p < .05$. ** indicates $p < .01$.

Supplementary Table 10. **Multiple Regression model with criterion and NUL predicting CEA with Study 1 sample.** While the overall model is significant and the individual zero-order correlations between CEA and criterion and NUL are significant, NUL no longer predicts CEA when controlling for criterion.

Predictor	*b*	*b* 95% CI [LL, UL]	*beta*	*beta* 95% CI [LL, UL]	sr^2	sr^2 95% CI [LL, UL]	*r*	Fit
(Intercept)	0.40**	[0.35, 0.46]						
c	-0.33**	[-0.36, -0.31]	-0.91	[-0.97, -0.86]	.77	[.70, .84]	-.92**	
AoA	0.00	[-0.00, 0.01]	0.02	[-0.04, 0.07]	.00	[-.00, .00]	.27**	
								R^2 = .845**<br>95% CI[.81,.87]

Note. * indicates $p < .05$. ** indicates $p < .01$.

Supplementary Table 11. **Multiple Regression model with criterion and AoA predicting CEA with Study 1 sample.** Similar to NUL, AoA no longer predicts CEA when controlling for criterion.

Predictor	b	b 95% CI [LL, UL]	$beta$	$beta$ 95% CI [LL, UL]	sr^2	sr^2 95% CI [LL, UL]	r	Fit
(Intercept)	0.58**	[0.52, 0.63]						
c	-0.45**	[-0.49, -0.41]	-1.11	[-1.22, -1.00]	.37	[.29, .45]	-.86**	
NUL	-0.02**	[-0.02, -0.01]	-0.29	[-0.40, -0.18]	.03	[.01, .05]	.63**	
								R^2 = .767**<br>95% CI[.72,.80]

Note. * indicates $p < .05$. ** indicates $p < .01$.

Supplementary Table 12. **Multiple Regression model with criterion and NUL predicting CEA with Study 2 sample.** Replicating results using Study 1 sample, NUL no longer predicts CEA when controlling for criterion.

Predictor	*b*	*b* 95% CI [LL, UL]	*beta*	*beta* 95% CI [LL, UL]	sr^2	sr^2 95% CI [LL, UL]	*r*	Fit
(Intercept)	0.43**	[0.35, 0.51]						
c	-0.34**	[-0.36, -0.31]	-0.86	[-0.92, -0.80]	.72	[.66, .78]	-.86**	
AoA	0.00	[-0.01, 0.01]	0.00	[-0.06, 0.06]	.00	[-.00, .00]	.14*	
								R^2 = .742**<br>95% CI[.69,.78]

Note. * indicates $p < .05$. ** indicates $p < .01$.

Supplementary Table 13. **Multiple Regression model with criterion and AoA predicting CEA with Study 2 sample.** Replicating results using Study 1 sample, AoA no longer predicts CEA when controlling for criterion.

	d'	*c*
MLU	. 073<br>[-.03, .18]	. 942**<br>[.93, .96]
SA	.724**<br>[.67, .78]	-. 208**<br>[-.31, -.10]
CEA	.508**<br>[.42, .59]	-.872**<br>[-.89, -.85]

Note. * indicates $p < .05$. ** indicates $p < .01$.

Supplementary Table 14. **Spearman correlations between SDT metrics and emotion segmentation metrics using integrative sample (*N*=381).** With larger combined sample size, MLU was still not correlated with sensitivity. Other emotion segmentation metrics correlated with both signal detection metrics in expected directions.

	d'	*c*
NUL	-.001<br>[-.11, .10]	-.876**<br>[-.91, -.84]
AoA	.057<br>[-.04, .16]	-. 188**<br>[-.28, -.09]

Note. * indicates p < .05. ** indicates p < .01.

Supplementary Table 15. **Spearman correlations between SDT metrics and active emotion vocabulary metrics using integrative sample (*N*=381).** Neither active emotion vocabulary metrics correlated with sensitivity, but both still significantly correlated with criterion in the expected direction.

Predictor	*b*	*b* 95% CI [LL, UL]	sr^2	sr^2 95% CI [LL, UL]	Fit
(Intercept)	0.45**	[0.43, 0.48]			
c	-0.43**	[-0.46, -0.39]	.32	[.25, .38]	
NUL	-0.01**	[-0.02, -0.01]	.01	[.00, .03]	
SampleMarkerS3	0.01	[-0.01, 0.02]	.00	[-.00, .00]	
					R^2 = .761**
					95% CI[.72,.79]

Note. * indicates p < .05. ** indicates p < .01.

Supplementary Table 16. **Multiple Regression model with criterion and NUL predicting CEA with integrative sample.** With the integrative sample, NUL only marginally predicts CEA when controlling for criterion.

Predictor	b	b 95% CI [LL, UL]	sr^2	sr^2 95% CI [LL, UL]	Fit
(Intercept)	0.47**	[0.44, 0.50]			
c	-0.33**	[-0.35, -0.31]	.71	[.66, .77]	
AoA	0.00	[-0.01, 0.01]	.00	[-.00, .00]	
SampleMarkerS3	0.00	[-0.01, 0.02]	.00	[-.00, .00]	
					R^2 = .746**<br>95% CI[.70,.78]

Note. * indicates $p < .05$. ** indicates $p < .01$.

Supplementary Table 17. **Multiple Regression model with criterion and AoA predicting CEA with integrative sample.** Replicating results using Study 1 and Study 2 sample, AoA no longer predicts CEA when controlling for criterion.

Supplementary Note 7. Exploratory analysis on emotion labels and segmentation frequency

In Study 1, the videos we selected seemed to be disproportionally featuring the emotion of anger (see main text Fig.4). It was possible that the frequency of these anger segmentations was not stimulus-driven, but rather was due to the accessibility of anger as a category. Indeed, prior literature suggests that anger, given its relation to threats, may be processed with higher priority and associated with attentional bias over emotions, particularly those that are positive[22-25]. Such perceptual bias may render segmentations for anger to be more frequent, because these instances appear as more "obvious." To explore whether anger is disproportionately featured in the spontaneous segmentations of participants, we computed the frequency of generated emotion labels and obtained the top five most generated labels for each video. We also computed the segmentation frequency of each video averaged across participants to investigate the effect of anger on segmentation performance.

Results showed that for Study 1 stimuli, 4 out of 6 emotional videos (*Selena*, *Love Rosie*, *Spotlight*, *Before Sunset*) have anger/angry in the top 5 most generated labels (Supplementary Figure 7). Among those 4 videos, 3 of them also have the highest average frequency of segmentation across subjects out of all videos (Supplementary Figure 8). For Study 2 stimuli, only 2 out of 9 videos (*Unrest*, *Minding the Gap_2*) have anger/angry in the top 5 labels (Supplementary Figure 9). These two videos respectively have the second and third highest average frequency of segmentation across subjects (Supplementary Figure 10).

Results from this exploratory analysis suggest a potential link between anger and segmentation behaviors. However, this link seems to be mitigated in Study 2 where more variation in emotional stimuli is included. Further research is needed to explore links between specific emotion categories, stimulus content, and the frequency of segmentation.

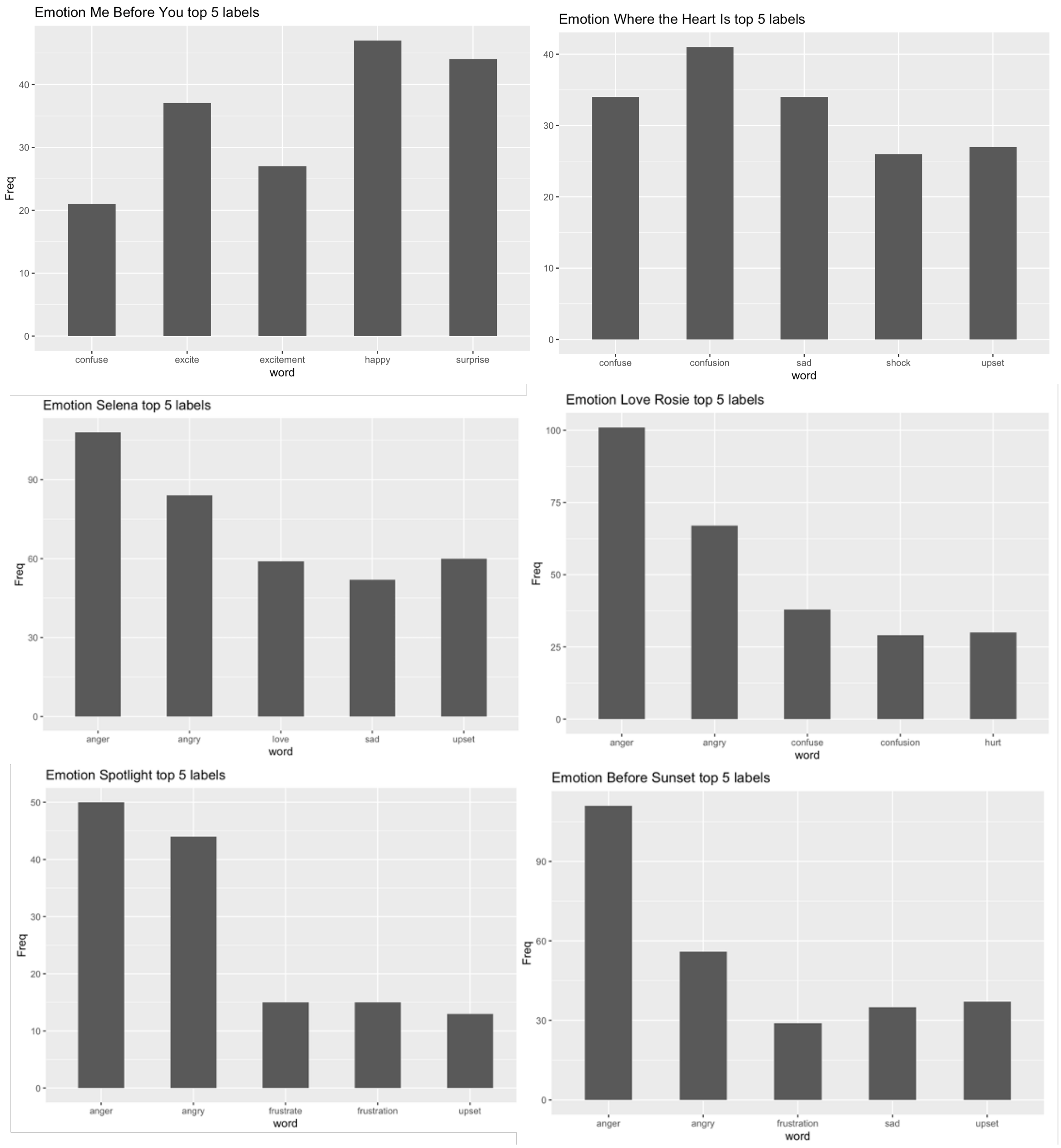

Supplementary Figure 7. Top five most frequently generated labels across participants in Study 1 stimuli. Among the 6 emotional stimuli, 4 (middle left and right, bottom left and right) had labels such as "anger" and "angry" generated the most.

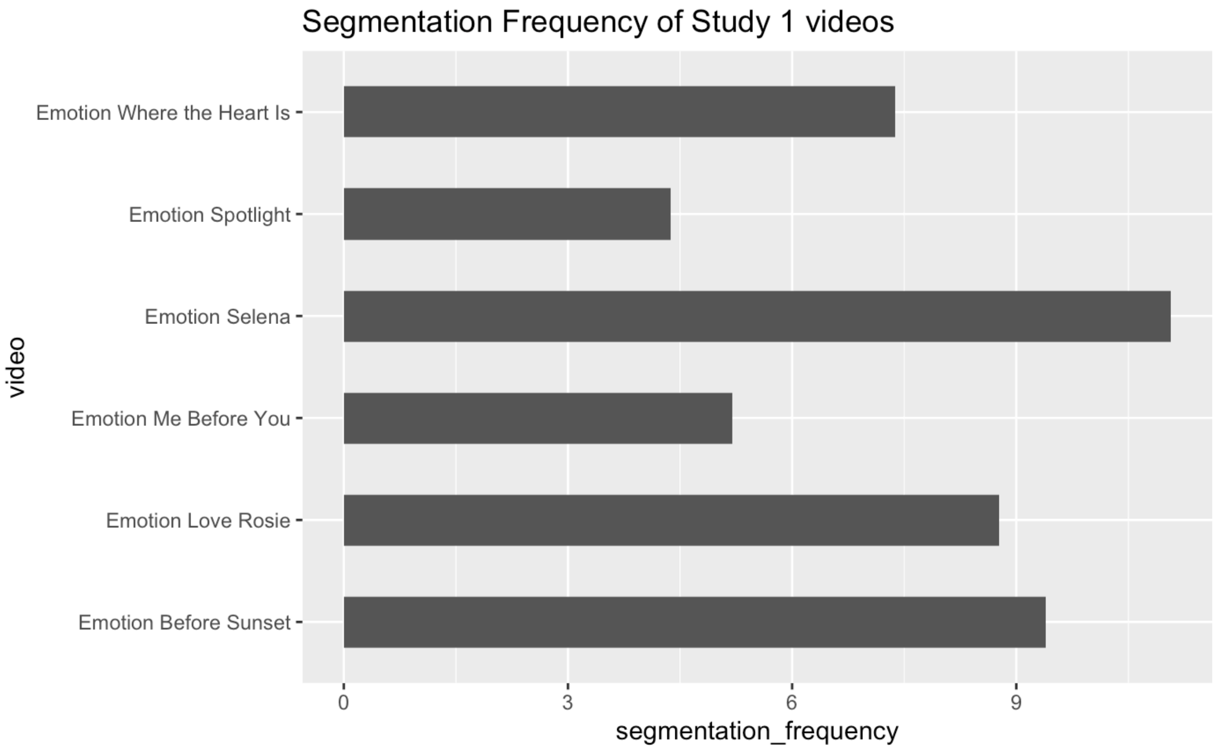

Supplementary Figure 8. **Segmentation frequency of emotional videos in Study 1.** Among the 6 videos, all three videos with the top three highest frequency of segmentation (*Selena*, *Love Rosie*, *Before Sunset*) also mainly feature anger.

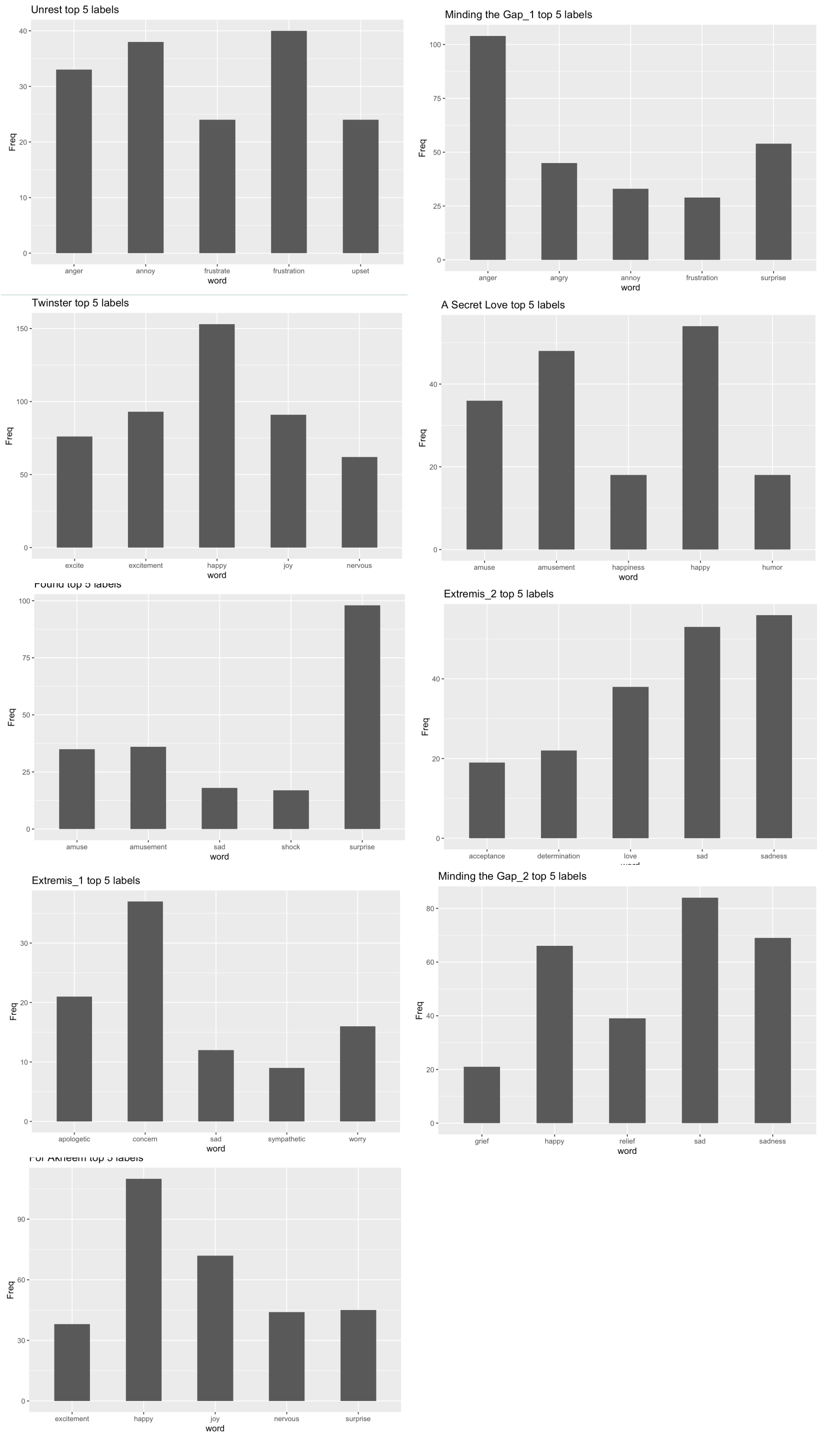

Supplementary Figure 9. **Top five most frequently generated labels across participants in Study 1 stimuli.** Among the 9 emotional stimuli, 2 (Top right and left) had labels such as "anger" and "angry" generated the most.

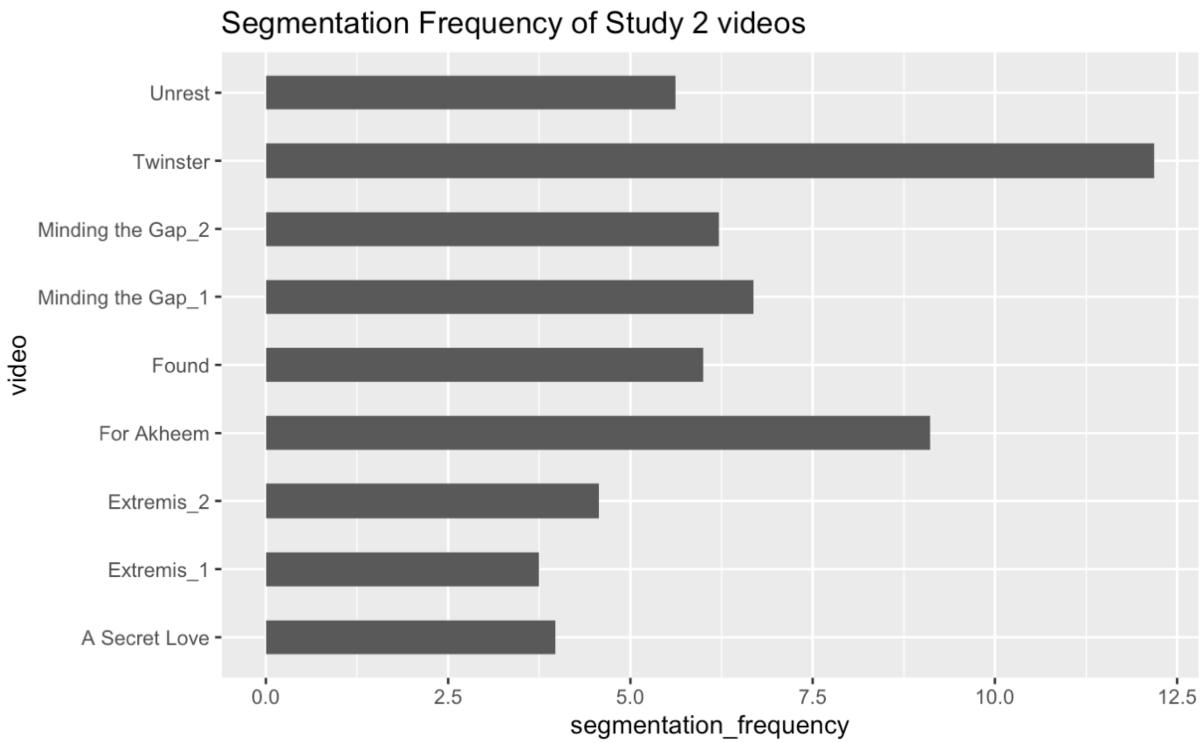

Supplementary Figure 10. **Segmentation frequency of emotional videos in Study 2.** Among the 9 videos, one of the three videos from the top three highest frequency of segmentation (Minding the Gap_1) also mainly features anger.

Supplementary references

1. Raji, S. & de Melo, G. What Sparks Joy: The AffectVec Emotion Database. in *Proceedings of The Web Conference 2020* 2991–2997 (Association for Computing Machinery, 2020).
2. Brooks, J. A. & Freeman, J. Conceptual knowledge predicts the representational structure of facial emotion perception. *Nat. Hum. Behav.* **2**, 581-591 (2018)
3. Brooks, J. A., Chikazoe, J., Sadato, N. & Freeman, J. B. The neural representation of facial-emotion categories reflects conceptual structure. *Proc. Natl. Acad. Sci.* **116**, 15861–15870 (2019).
4. Le Mau, T. *et al.* Professional actors demonstrate variability, not stereotypical expressions, when portraying emotional states in photographs. *Nat. Commun.* **12**, 5037 (2021).
5. Cordaro, D. T. *et al.* The recognition of 18 facial-bodily expressions across nine cultures. *Emotion* **20**, 1292–1300 (2020).
6. Cowen, A. S., Elfenbein, H. A., Laukka, P. & Keltner, D. Mapping 24 emotions conveyed by brief human vocalization. *Am. Psychol.* **74**, 698–712 (2019).
7. Cowen, A. S. & Keltner, D. Self-report captures 27 distinct categories of emotion bridged by continuous gradients. *Proc. Natl. Acad. Sci.* **114**, (2017).
8. Cowen, A. S. & Keltner, D. What the face displays: Mapping 28 emotions conveyed by naturalistic expression. *Am. Psychol.* **75**, 349–364 (2020).
9. Cowen, A. S., Laukka, P., Elfenbein, H. A., Liu, R. & Keltner, D. The primacy of categories in the recognition of 12 emotions in speech prosody across two cultures. *Nat. Hum. Behav.* **3**, 369–382 (2019).
10. Zaki, J. Integrating Empathy and Interpersonal Emotion Regulation. *Annu. Rev. Psychol.* **71**, 517–540 (2020).

11. Vine, V., Boyd, R. L. & Pennebaker, J. W. Natural emotion vocabularies as windows on distress and well-being. *Nat. Commun.* **11**, 4525 (2020).

12. Lucas, R. E. Reevaluating the Strengths and Weaknesses of Self- Report Measures of Subjective Well-Being. in *Handbook of well-being* (eds. Diener, E., Oishi, S. & Tay, L.) (DEF Publishers, 2018)

13. Martin, W. W. & Rovira, M. Signal Detection Theory: Its Implications for Social Psychology. *Pers Soc Psychol Bull* **7**, 232–239 (1981).

14. Stanislaw, H. & Todorov, N. Calculation of signal detection theory measures. *Behavior Research Methods, Instruments, & Computers* **31**, 137–149 (1999).

15. Macmillan, N. A. & Creelman, C. D. *Detection Theory: A User's Guide*. (Taylor & Francis Group, 2004).

16. Hautus, M. J. Corrections for extreme proportions and their biasing effects on estimated values ofd′. *Behavior Research Methods, Instruments, & Computers* **27**, 46–51 (1995).

17. Hayes, A. F. & Coutts, J. J. Use Omega Rather than Cronbach's Alpha for Estimating Reliability. But…. *Communication Methods and Measures* **14**, 1–24 (2020).

18. Karmon-Presser, A., Sheppes, G. & Meiran, N. How does it 'feel'? A signal detection approach to feeling generation. *Emotion* **18**, 94–115 (2018).

19. Lord, F. M., Novick, M. R. & Birnbaum, A. *Statistical theories of mental test scores*. (Addison-Wesley, 1968).

20. Cronbach, L. J. & Furby, L. How we should measure 'change': Or should we? *Psychological Bulletin* **74**, 68–80 (1970).

21. Lynn, S. K. & Barrett, L. F. "UTILIZING" SIGNAL DETECTION THEORY. *Psychol Sci* **25**, 1663–1673 (2014).

22. Fox, E., Russo, R. & Dutton, K. Attentional bias for threat: Evidence for delayed disengagement from emotional faces. *Cognition and Emotion* **16**, 355–379 (2002).

23. Ince, S. *et al.* Subcortical contributions to salience network functioning during negative emotional processing. *NeuroImage* **270**, 119964 (2023).

24. Feldmann-Wüstefeld, T., Schmidt-Daffy, M. & Schubö, A. Neural evidence for the threat detection advantage: Differential attention allocation to angry and happy faces. *Psychophysiology* **48**, 697–707 (2011).

25. Norris, C. J. The negativity bias, revisited: Evidence from neuroscience measures and an individual differences approach. *Soc Neurosci* **16**, 68–82 (2021).
