# Supplementary material for: Emotional event perception is related to lexical complexity and emotion knowledge
Source: Commun Psychol. 2023 Dec 18;1:45. doi: 10.1038/s44271-023-00039-4 (PMC11332234; doi:10.1038/s44271-023-00039-4)
Supplement: Supplementary file 3 — Reporting Summary [file 44271_2023_39_MOESM3_ESM.pdf]

nature portfolio

Corresponding author(s): Dr.Maria Gendron

Last updated by author(s): 08/17/2023

Reporting Summary

Nature Portfolio wishes to improve the reproducibility of the work that we publish. This form provides structure for consistency and transparency in reporting. For further information on Nature Portfolio policies, see our Editorial Policies and the Editorial Policy Checklist.

Statistics

For all statistical analyses, confirm that the following items are present in the figure legend, table legend, main text, or Methods section.

n/a	Confirmed	
☐	☒	The exact sample size (*n*) for each experimental group/condition, given as a discrete number and unit of measurement
☐	☒	A statement on whether measurements were taken from distinct samples or whether the same sample was measured repeatedly
☐	☒	The statistical test(s) used AND whether they are one- or two-sided<br>*Only common tests should be described solely by name; describe more complex techniques in the Methods section.*
☒	☐	A description of all covariates tested
☐	☒	A description of any assumptions or corrections, such as tests of normality and adjustment for multiple comparisons
☐	☒	A full description of the statistical parameters including central tendency (e.g. means) or other basic estimates (e.g. regression coefficient) AND variation (e.g. standard deviation) or associated estimates of uncertainty (e.g. confidence intervals)
☐	☒	For null hypothesis testing, the test statistic (e.g. *F*, *t*, *r*) with confidence intervals, effect sizes, degrees of freedom and *P* value noted<br>*Give P values as exact values whenever suitable.*
☒	☐	For Bayesian analysis, information on the choice of priors and Markov chain Monte Carlo settings
☒	☐	For hierarchical and complex designs, identification of the appropriate level for tests and full reporting of outcomes
☐	☒	Estimates of effect sizes (e.g. Cohen's *d*, Pearson's *r*), indicating how they were calculated

Our web collection on statistics for biologists contains articles on many of the points above.

Software and code

Policy information about availability of computer code

Data collection	Data were collected via Qualtrics and a custom-built platform across two sessions which were spaced at least one week a part.
Data analysis	Data were analyzed using open source software R (version 4.3.0) and the open-source linguistic analysis tool TAALES (version 2.2). All analysis scripts and packages relied upon are available in OSF repository: https://osf.io/zsu7t/?view_only=90f62ea1056c4b4e995514d99fe59e3a.

For manuscripts utilizing custom algorithms or software that are central to the research but not yet described in published literature, software must be made available to editors and reviewers. We strongly encourage code deposition in a community repository (e.g. GitHub). See the Nature Portfolio guidelines for submitting code & software for further information.

Data

Policy information about availability of data

All manuscripts must include a data availability statement. This statement should provide the following information, where applicable:

- Accession codes, unique identifiers, or web links for publicly available datasets
- A description of any restrictions on data availability
- For clinical datasets or third party data, please ensure that the statement adheres to our policy

The quantitative and qualitative data (deidentified) collected and analyzed for Studies 1–3 are available in the OSF repository at https://osf.io/zsu7t/?view_only=90f62ea1056c4b4e995514d99fe59e3a.

Human research participants

Policy information about studies involving human research participants and Sex and Gender in Research.

Reporting on sex and gender	Samples were collected on Prolific using the "balanced" sample feature that recruits the same number of participants across sex, but due to attrition across the two sessions the final samples were not balanced. Our analyses do not constrain to one sex or gender and sex and/or gender was not further considered in the study design. Information about sex was provided by participants. This information is provided in the source data and summarized in the methods.
Population characteristics	Research samples were convenience samples recruited online and consisted of healthy adults who are native English speakers and were born and currently living in the United States. We set such criteria to ensure consistency in participants' cultural and linguistic background, as different backgrounds may impact emotion perception, the focus of the study. We did not use representative samples, but samples are balanced by sex.
Recruitment	Participants were recruited via Prolific. There may be self-selection bias considering only workers of this online platform with access to the Internet were able to participate in the study.
Ethics oversight	Yale University Institutional Review Board, IRB# 2000026863

Note that full information on the approval of the study protocol must also be provided in the manuscript.

Field-specific reporting

Please select the one below that is the best fit for your research. If you are not sure, read the appropriate sections before making your selection.

☐ Life sciences ☒ Behavioural & social sciences ☐ Ecological, evolutionary & environmental sciences

For a reference copy of the document with all sections, see nature.com/documents/nr-reporting-summary-flat.pdf

Behavioural & social sciences study design

All studies must disclose on these points even when the disclosure is negative.

Study description	All three studies are behavioral studies with both quantitative (timestamp of pausing video, Likert-type ratings) and qualitative data (emotion labels, responses to open-ended questions) collected.
Research sample	Study 1 (mean age=32.18) consists of 114 male participants, 98 female participants, 10 participants who identified as non-binary; 145 identified as White, 22 identified as Black or African American, 19 identified as Asian, 11 identified as Hispanic or Latinx, 3 identified as American Indian or Alaskan Native, 21 identified as mixed race, and 1 identified as 'other'.<br><br>Study 2 (mean age = 35.24) consists of 111 male participants, 140 female participants, 9 participants who identified as non-binary or others; 194 identified as White, 17 identified as Black or African American, 11 identified as Asian, 12 identified as Hispanic or Latinx, 23 identified as mixed race, and 4 identified as 'other.<br><br>Study 3 (mean age = 34.94) consists of 53 male participants, 43 female participants, 5 participants who identified as non-binary or others; 67 identified as White, 11 identified as Black or African American, 5 identified as Asian, 5 identified as Hispanic or Latinx, 11 identified as mixed race, and 2 identified as 'other.'
Sampling strategy	For Study 1, the planned sample size was 260 participants but only 241 participants were successfully recruited and retained across sessions on Prolific. Data collection was stopped early after multiple attempts to recruit a larger sample and a temporary technical issue with our custom platform leading to data loss. For Study 2 (preregistered), we aimed to recruit N=260, the sample size at which the effect size of correlations stabilize. A sensitivity analysis showed that we were statistically powered to detect the effect size of 0.214 (α = 0.008 for multiple comparison, power = 0.8), which fell below the range of previous effect sizes of Study 1 (rs = 0.26 - 0.49), For Study 3, we aimed to recruit N=101, the sample size which, according to the power analysis, is sufficiently powered to detect the estimation of an ICC of 0.7 (Precision = 0.1; α = 0.05, power = 0.8). We oversampled N=120 to account for attrition.
Data collection	Data collection was completed online via computer. Participants completed tasks without the presence of the researcher.
Timing	Study 1 wave 1: 3/17/2021-4/6/2021<br>Study 1 wave 2: 5/29/2021-7/4/2021<br>Study 1 wave 3: 7/23/2021-8/28/2021<br>Study 2 wave 1: 12/9/2021-1/22/2022<br>Study 2 wave 2: 1/7/2022-1/25/2022<br>Study 2 wave 3: 2/2/2022-2/15/2022<br>Study 2 wave 4: 2/19/2022-2/26/2022<br>Study 3 wave 1: 5/10/2022-5/22/2022

	Study 3 wave 2: 6/2/2022-6/17/2022<br>Study 3 wave 3: 6/18/2022-6/28/2022
Data exclusions	Participants who completed both sessions but who failed two attention checks and who were low-effort (i.e., did not segment all videos) were excluded.
Non-participation	Participants who consented to the study but did not return for the second session were not included in the final samples reported in the manuscript. Attrition across sessions was approximately 15%.
Randomization	There were no conditions and all participants completed all tasks to be retained in the final sample reported. For Studies 1 and 2, the order by which participants completed the two sessions was counterbalanced.

Reporting for specific materials, systems and methods

We require information from authors about some types of materials, experimental systems and methods used in many studies. Here, indicate whether each material, system or method listed is relevant to your study. If you are not sure if a list item applies to your research, read the appropriate section before selecting a response.

Materials & experimental systems

n/a	Involved in the study
☒	☐ Antibodies
☒	☐ Eukaryotic cell lines
☒	☐ Palaeontology and archaeology
☒	☐ Animals and other organisms
☒	☐ Clinical data
☒	☐ Dual use research of concern

Methods

n/a	Involved in the study
☒	☐ ChIP-seq
☒	☐ Flow cytometry
☒	☐ MRI-based neuroimaging
